# In‐Liquid Micromanipulation via a Magnetic Microactuator for Multitasking

**DOI:** 10.1002/smsc.202500010

**Published:** 2025-03-30

**Authors:** Dineshkumar Loganathan, Chia‐Hsin Cheng, Po‐Wei Wei, Chia‐Yuan Chen

**Affiliations:** ^1^ Department of Mechanical Engineering National Cheng Kung University Tainan 701 Taiwan

**Keywords:** fluid flow conveyance, magnetic microactuator, microassembly, micromixing, particle manipulation

## Abstract

Small‐scale actuators capable of performing multiple tasks are crucial for the advancement of microfluidic technologies. These actuators enable high‐throughput operations and support integrated solutions across a wide range of applications. In this study, a multipurpose magnetic microactuator (MMA) is developed with two pairs of magnetic arms controlled externally through a custom‐built electromagnetic system. To enhance navigational precision, two circular magnetic sections named “mobility components” are integrated into the MMA's design. The multitasking capability of the MMA is demonstrated through distinct applications, including particle manipulation, microassembly, micromixing, and flow conveyance. In particle manipulation, the MMA is controlled to grasp a total of eight particles from different locations in a single cycle within 46 s. During the assembly process, two 2D planar micro‐objects are sequentially loaded, transported, and assembled in the designated assembly unit. For fluid flow control, the distinct motions of the MMA are observed to enhance the mixing performance with an efficiency of 65% within 20 s. In addition, the dye conveyance efficiency is observed to reach 85% for the MMA's navigational distances of 10 mm in 30 s. These results demonstrate the MMA's capacity for synergistic multitasking with increased throughput, establishing it as a foundation for future microfluidic actuators.

## Graphical abstract

A multipurpose magnetic microactuator with two pairs of magnetic arms and mobility components is developed to perform multiple tasks, seamlessly combining adaptability and precision for complex microfluidic operations. This design enables synchronized and differentiated motions, allowing the manipulation of microparticles of various sizes and 2D planar objects with high throughput, offering promising solutions for biomedical and lab‐on‐a‐chip applications.

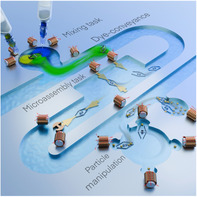

## 1 Introduction

Small‐scale actuators, typically ranging in size from a few millimeters to hundreds of micrometers, are essential for precise, adaptable, and space‐efficient manipulation across various applications. In particular, these devices are indispensable for tasks such as small‐scale object handling, assembly processes, and fluid flow management within confined spaces. In liquid environments, their importance has been particularly emphasized due to their ability to perform complex operations in constrained settings, such as within microchannels or biomedical systems, where conventional mechanical methods face limitations.^[^ ^1^ ^]^ Meanwhile, several approaches and techniques have been proposed to control and actuate these devices, including acoustic,^[^ ^2^ ^]^ electrostatic,^[^ ^3^ ^]^ piezoelectric,^[^ ^4^ ^]^ thermal,^[^ ^5^ ^]^ and mechanical methods.^[^ ^6^ ^]^ For example, piezoelectric actuation was reported to utilize the deformation of specialized materials under electrical stimulation,^[^ ^7^ ^]^ while acoustic actuation employed ultrasonic waves to generate localized forces for precision manipulation.^[^ ^8^ ^]^ However, integrating multiple control components into these actuators was reported to increase structural complexity and reduce efficiency in liquid environments.^[^ ^9^ ^]^ For example, a multimode hybrid actuator was proposed to manipulate and assemble micro and millimeter‐scale objects of varying sizes.^[^ ^10^ ^]^ This actuator integrated four subassembly components, including a tool changer, pneumatic cylinder, vacuum gripper, and mechanical gripper, into their structure. Though this design demonstrated the potential to perform diverse tasks such as particle manipulation and assembly, the intricate architecture may significantly increase the fabrication challenges and limit the operational efficiency in confined environments. To address these limitations, it is crucial to develop the actuator with simplified designs and streamlined control approaches. By minimizing the integration of excessive control components, such designs could reduce structural complexity and enhance operational efficiency in constrained spaces. Furthermore, these advancements are anticipated to simplify fabrication processes and broaden the applicability of small‐scale actuators for diverse tasks, particularly in liquid environments where traditional complex designs face significant challenges.

A control system utilizing magnetic fields^[^ ^11^, ^12^, ^13^, ^14^, ^15^, ^16^, ^17^, ^18^, ^19^, ^20^, ^21^, ^22^, ^23^, ^24^ ^]^ to manipulate actuators can effectively address the identified limitations, offering distinct advantages such as untethered operation, noninvasive control, and precise manipulation with faster responsiveness. In particular, the externally generated magnetic field can be employed to control both the movements of the actuator's arms and the navigation of the entire actuator, thereby eliminating the need for separate subcomponents dedicated to these functions. Subsequently, this approach can pave the way for achieving a simplified actuator design with reduced structural complexity. For instance, a mobile magnetic microactuator (MMA) with embedded permanent magnets was designed to manipulate microparticles under the actuation of the external magnetic field.^[^ ^25^ ^]^ In the same work, the magnetic field was further employed to independently control the navigation of multiple actuators, enabling the assembly process by precisely positioning the grasped particles. Although several similar magnetic actuators have been developed,^[^ ^26^, ^27^, ^28^ ^]^ enhancing the throughput by manipulating multiple objects in a single working cycle to reduce processing time and improve overall performance remains a significant challenge. Therefore, this improvement of handling more objects in a single cycle is in high demand, as they would enable efficient and scalable solutions for a wide range of applications that can further expand the potential of magnetic actuation methods in microfluidic and clinical settings.

The ability of the microactuator to perform multiple tasks, including particle or object handling, microassembly, and fluid flow manipulation through a single architectural design, is a key advantage for advancing microfluidic systems. However, the shift from handling particles to manipulating microsized objects presents unique challenges, primarily stemming from differences in size, weight, and interaction forces. In particular, the particles that are smaller and lighter require precise control and minimal force to prevent damage or displacement, whereas microsized objects, due to their larger dimensions and weight, necessitate greater gripping force and stability during manipulation. Though several microactuators have been proposed, they were primarily employed for either micro/nanoscale particle manipulation or 2D/3D microscale object handling.^[^ ^29^ ^]^ However, integrating the capability to perform both tasks within the same structural design remains complex and underexplored. For instance, a flower‐shaped magnetic actuator with rigid and flexible petals like magnetic arms was developed to manipulate more than one particle per working cycle.^[^ ^30^ ^]^ However, it has been reported that manipulating planar objects using this structure is challenging due to structural limitations, as the overlap and interference of adjacent petal‐shaped arms hinder effective grasping. In addition to these primary tasks (such as particle and object handling abilities), actuators capable of effectively manipulating fluid can eliminate the need for integrated components such as pumps and valves within microchannels. This reduction simplifies the overall system design, minimizes fabrication complexity, and enhances operational efficiency. Furthermore, integrating fluid manipulation capabilities into the same actuator architecture can provide synergistic effects that improve the efficiency of particle or object handling. For instance, the ability to generate dynamic flow patterns during mixing can aid in the dispersion or distribution of particles in a controlled manner so that the targeted particle can be grasped from a group. By integrating these capabilities into a versatile design, the actuator can execute a range of functions without the need for additional specialized systems. Such microactuators are in high demand for applications requiring efficient, scalable, and integrated solutions in microfluidic systems, where adaptability and multifunctionality are critical.

**Figure 1:**
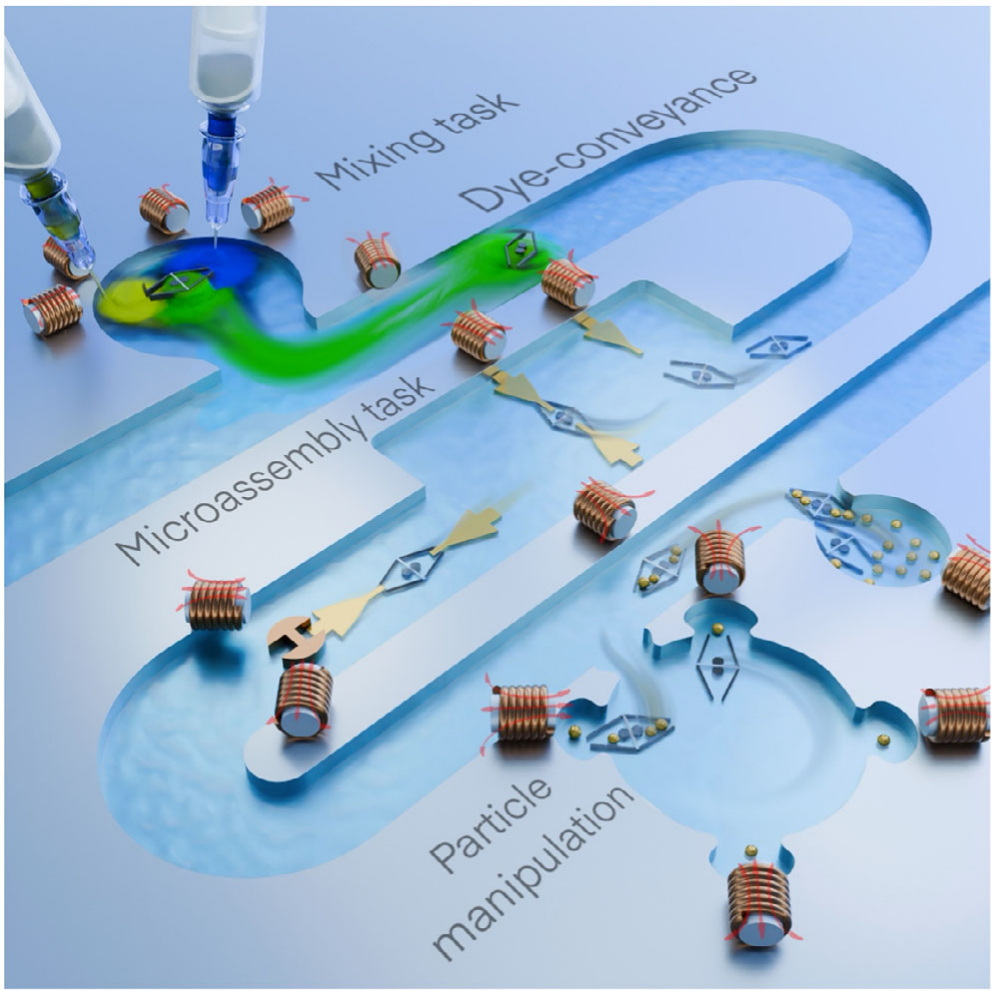
A schematic representation of the presented MMA illustrates its application in four distinct tasks, including microparticle grasping, 2D planar object assembly, flow conveyance, and micromixing. Further, it is shown that the mentioned tasks were conducted within a microfluidic environment and were achieved under the influence of an external magnetic field generated by custom‐built electromagnetic coils.

In this study, a MMA was developed and demonstrated to perform multiple tasks, including particle (microsized spherical‐shaped) handling, 2D planar micro‐object assembly, and fluid flow manipulation such as micromixing and microflow conveyance, as shown in **Figure** **1**. To enhance the navigational capabilities of the MMA, a dedicated mobility component was integrated into its architecture, enabling precise positioning and manipulation within confined environments. Additionally, the MMA was shown to execute high‐throughput operations, handling multiple particles or planar objects within a single working cycle. The presented design highlights the potential of the MMA as a versatile and efficient tool for microfluidic applications, addressing the challenges of multitasking and throughput in a single, integrated platform. The following sections provide a detailed explanation of the MMA's design, operational strategies, and performance outcomes across various tasks.

## 2 Results and Discussion

**Figure 2:**
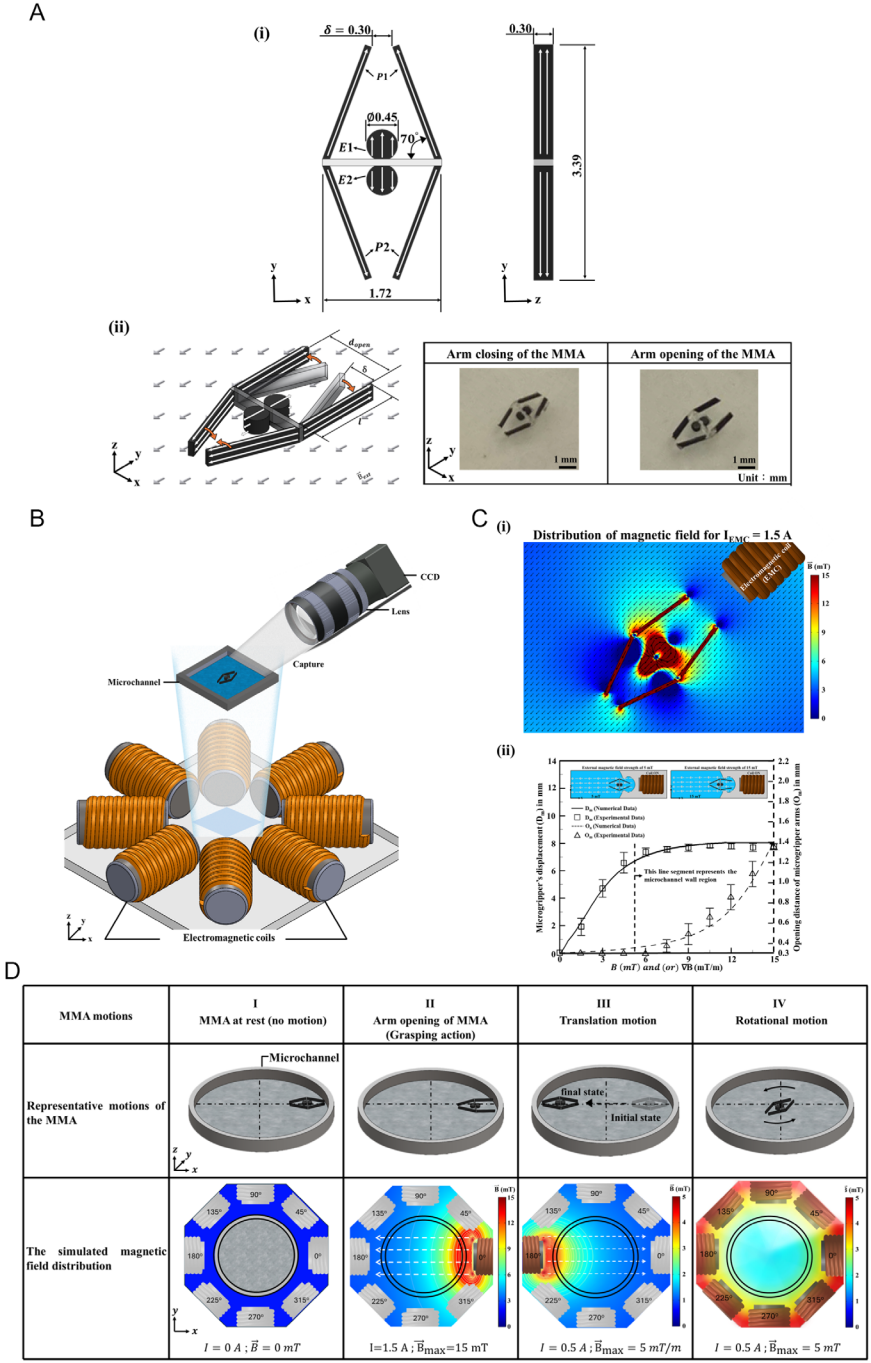
Schematic representation and operational analysis of the proposed MMA. A) Structural design of the MMA, illustrating the geometric dimensions in three orthogonal views (i). The mechanism of arm actuation is highlighted, demonstrating the closing and opening actions of the microgripper under an applied magnetic field (ii). Optical images depicting the arm's closed and open states. B) Experimental setup for MMA operation, showing the integration of electromagnetic coils surrounding a microchannel. A CCD camera with a lens system was employed for real‐time observation and data capture of MMA motion. C) Analysis of the magnetic field distribution and microgripper displacement. (i) Simulated distribution of the magnetic field intensity across the MMA structure for an input current of 1.5 A. Further, the magnetic field strength of 15 mT can be seen to open up the arms for grasping. (ii) A magnetic field of 5 mT m^−1^ was observed to propel the MMA to a distance of 8 mm. In addition, the MMA arms were observed to open up at the magnetic field of 5 mT and reached their maximum opening distance of 1.4 mm at the magnetic field of 15 mT. The inset shows the relationship between the magnetic field intensity and arm‐opening displacement. The error bars represent a unit standard deviation in both directions, calculated from the results of three independent experiments. D) Functional demonstration of the MMA's motion capabilities. Together with the resting state of MMA (I), three distinct modes of motion are illustrated, including grasping motion via arm opening (II), translational motion along a defined path (III), and rotational motion around the central axis (IV). Each mode is supported by the corresponding simulated magnetic field distribution, highlighting the variations in field intensity and direction for specific MMA actions.

The presented MMA was designed with two pairs of actuator (gripping) arms (a total of four actuator arms), as shown in **Figure** **2**A(i). Further, two circular components, referred to as the “eyes” (or the mobility components) of the actuator, were positioned at the center of the MMA structure and were employed to control its movements. A detailed fabrication process of the MMA is illustrated in Figure S1, Supporting Information. To enable the functionality of the MMA, all four arms and the two eyes were magnetized, and their respective magnetization directions are shown in Figure 2A(i). It should be noted that the two pairs of the actuator arms (P1 and P2) and the eyes (E1 and E2) were magnetized in opposite directions to each other. This magnetization profile was specifically chosen to ensure the sequential grasping of objects on either side of the MMA. For instance, when the pair of arms P1 was controlled (by an external magnetic field $\left(\vec{B_{\text{ext}}}\right)$) to open up for releasing the object, the other pair P2 can simultaneously undergo a closing action for grasping the object due to the considered magnetization profile. Meanwhile, the magnetization direction for the eyes was chosen to be aligned with the magnetization direction of the respective adjacent arm pair. For example, the magnetization direction of the eye E1 can be seen to be aligned with the magnetization direction of the pair of arms P1. The magnetization profile was carefully selected to ensure precise control over the MMA's movements, thereby enabling its effective functioning. Notably, the default distance between any pair of actuator arms was 0.3 mm $\left(\vec{B_{\text{ext}}}=0\right)$ (Figure 2A(i)), ensuring that the MMA was well‐suited for grasping objects or particles with sizes of 0.3 mm or larger. Upon being subjected to the maximum value of the external magnetic field $\left(\vec{B_{\text{max}}}\right)$, the actuator arms pair P1 can be seen to be opened up at a distance 1.4 mm (maximum) apart (Figure 2A(ii)). Therefore, it was ensured that the presented MMA could grasp or release objects or particles within the size range of 0.3 mm < *d*_p_ < 1.4 mm (where “*d*_p_” denotes the particle diameter, Figure S2, Supporting Information). The optical image of the actuator with one of its arm pairs opened is also shown in Figure 2A(ii). In order to actuate and control the motions of the MMA, a custom‐built eight‐coil electromagnetic system, as shown in Figure 2B, was employed. A detailed construction and assembly procedure for this external drive system is provided in the Experimental Section, “The Magnetic Drive System”. In addition to the mentioned magnetization direction of the actuator arms, selected external magnetic field factors such as strength and direction that can significantly affect the actuator arms’ opening and closing actions are discussed extensively in the subsequent sections.

### 2.1 The Motion Dynamics of the MMA

The precise control of MMA motions can directly impact their ability to handle, manipulate, and position small‐scale objects with high accuracy. Further, effective manipulation of the MMA requires a thorough comprehension of how the MMA's movement translates into functional actions, such as gripping and releasing from different tortuous locations inside the microenvironment, which is essential for applications ranging from microparticle manipulation to automation in microassembly processes. In this work, the authors analyzed the interplay between the design of the MMA and the motion dynamics induced by the external magnetic field. In addition to the magnetization profile of each magnetic section in the MMA, the strength and direction of the external magnetic field were observed to determine the motions of the MMA. To analyze the MMA motions upon being subjected to the external magnetic field, the resultant magnetic field distribution across the magnetic sections of the MMA was simulated and studied. For example, Figure S3(i), Supporting Information, shows the distribution of the resultant magnetic field that was simulated for the input current of 0.5 A. Here, the value of *B*_max_ can be seen to be 5 mT, which was distributed across all the magnetic sections of the MMA. Further, for the mentioned range of magnetic field (0–5 mT), the magnetic arms can be seen not to be deflected. That is, this simulation indicated that neither the arms were opened up nor closed further for the mentioned value of the input parameters. However, the displacement (or the movement) of the MMA was predicted, and it was further validated with the experimental results (shown in Figure S3(ii), Supporting Information). This figure further demonstrates the range of magnetic field values that were necessary to displace (translate) the MMA inside the microchannel. It can be seen that the existence of a minimum magnetic field gradient of 1.05 mT m^−1^ was required to displace the MMA of (*D*_m_ = 0.1 mm). Similarly, to displace the MMA at a distance of 8 mm, the required magnetic field gradient was predicted (and validated experimentally) to be 5 mT m^−1^. The inserted figure depicts the translational motion (along the *x*‐axis) of the MMA from the origin (*x* = 0) to near the wall of the microchannel (*x* = 8 mm) when the electromagnetic coil (EMC) was supplied with the input current of 0.5 A. In contrast to the mentioned MMA motion, the pair of actuator arms positioned near the EMC was predicted to open up (arm pair P1) when the EMC was simulated with the input current of 1.5 A (Figure 2C(i)). Further, the variation of the actuator arm opening distance for a range of external magnetic field values is shown in Figure 2C(ii). This figure illustrates that a minimum external magnetic field of over 5 mT was necessary to open the MMA arms when positioned adjacent to the microchannel wall. Similarly, the external magnetic field of 15 mT can be seen to completely open up the MMA arms (*O*_m_ = 1.4 mm, which was the maximum opening distance that the presented actuator arms could open). Further, the inserted figures illustrate the comparison of the opening action of the MMA that was positioned near the microchannel wall at different values of the external magnetic field (5 and 15 mT). Meanwhile, together with the two fundamental motions of the gripper‐based actuator, such as the closing and opening actions of the arms for grasping and releasing, respectively, the presented MMA was demonstrated with two navigational motions, such as translation and rotation. All the mentioned MMA motions are shown in Figure 2D. Further, the simulated strength of the external magnetic field and the direction that was required to achieve the respective motions of the MMA are shown in Figure 2D. As discussed, the simulated results indicated that a magnetic field strength of 15 mT was necessary to achieve the opening action of the actuator arm near the microchannel wall. This magnetic field strength was simulated using EMC with a current supply of 1.5 A. However, in order to navigate (translate or rotate) the MMA inside the microchannel, the simulation result suggested the existence of a minimum magnetic field of 1.05 mT m^−1^. For instance, to translate the MMA from one end of the microchannel to the other (along the *x*‐axis, from the positive to the negative side, as shown in Figure 2D (III, Translation motion)), the simulation results suggested that the EMC positioned nearer to the destination zone needed to be supplied with a current of 0.5 A, resulting in a maximum magnetic field gradient strength 5 mT m^−1^. For the rotational motion of the MMA, while the minimum magnetic field of 1.05 mT was adequate, the current supplied to the EMCs surrounding the MMA needs to adhere to a phase difference methodology. A detailed illustration of the “current phase difference method” is provided in Figure S5, Supporting Information. With the help of this comprehensive motion dynamics analysis, two critical factors can be noted that were essential for the effective functioning of the MMA. First, to displace (translate) the MMA from one location to the other inside the microchannel, a magnetic field gradient of at least 1.05 mT m^−1^ needed to exist. Second, to reveal the fundamental motion of the MMA, that is, the opening of the actuator arms, a magnetic field of 15 mT needs to be generated around the location of the MMA. Meanwhile, to reveal the simultaneous functionalities of the MMA, such as arm opening and navigational motion, the mentioned values (1.05–15 mT) of the externally applied magnetic field were observed to be a determining factor. For instance, while a field strength above 1.05 mT was observed to facilitate translational motion (the magnetic field gradient), achieving simultaneous arm opening necessitated an increase beyond 5 mT, with a maximum opening distance of 1.4 mm observed at 15 mT in proximity to the microchannel wall. These findings indicate that while translational motion can be initiated at lower magnetic field gradients, simultaneous navigation and arm actuation require a threshold field of 5 mT or higher. Further, this range of magnetic field values necessary for the effective operation of the presented MMA was consistent with those employed in previous studies^[^ ^31^ ^]^ on small‐scale magnetic devices designed for microfluidic applications, including particle manipulation, microassembly, and micromixing.

### 2.2 Different Types of Motions Executed by the MMA

The dynamic motion capabilities of small‐scale actuators are essential for precise manipulation and controlled interactions within microfluidic systems. As described, the MMA developed in this study was externally controlled to perform various motions, including translational movements in horizontal, vertical, and inclined directions; full rotational motion; and periodic swinging actions, as shown in **Figure** **3**A (Modes I–V). Furthermore, coordinated arm movements of MMA, comprising opening and closing actions, were employed to facilitate the targeted release and capture of particles or micro‐objects, as depicted in Figure 3A (Modes VI and VII). A detailed analysis of the displacements along the *x*‐ and *y*‐axes for each motion type was performed to characterize these motion capabilities, with the corresponding mathematical representations provided in Figure 3A. For instance, the *x*‐ and *y*‐axis displacements of the MMA during translational motion were expressed as $\left(x_{1}+\Delta x\right)$ and $y_{1}$, respectively. Here, $x_{1}$ represents the initial position of the MMA's center along the *x*‐axis, $y_{1}$ corresponds to the initial position along the *y*‐axis, and Δx denotes the horizontal displacement of the MMA. This translational motion along the *x*‐axis was achieved by activating the EMC positioned at 0°, highlighted in blue in Figure 3A. Similarly, vertical translational motion and inclined navigation were accomplished by activating EMCs positioned at 90° and 45°, respectively. Meanwhile, the full rotational motion was achieved through the current‐phase difference method, which sequentially activated the EMCs with a phase offset. Further, the swinging motion was generated by selectively activating EMCs positioned at 135°, 90°, and 45°, with the associated displacement equations shown in Figure 3A (Mode V). The spatiotemporal behavior of the MMA during these motions was further analyzed and visualized in Figure 3B, which illustrates the trajectories of the MMA along the *x*‐ and *y*‐axes. For example, Figure 3B(i) demonstrates the translational motion along the *x*‐axis, where the MMA traversed a distance of 7 mm within 2 s while maintaining negligible displacement along the *y*‐axis. Similarly, Figure 3B(ii),(iii) depicts the spatiotemporal patterns for vertical translation and inclined navigation, respectively. The displacement profiles corresponding to full rotational motion (Mode V) and swinging motion (Mode VI) are presented in Figure 3B(iv),(v). In the case of swinging motion, the periodic displacements along the *x*‐axis were observed to be significantly greater than those along the *y*‐axis, with minimal variations observed in the latter. This behavior was attributed to the initial alignment of the MMA along the *y*‐axis during the swinging motion. Conversely, if the MMA was initially aligned along the *x*‐axis, then the displacement values along the *x* and *y* axes would be reversed. Additionally, the *x* and *y* axes displacement of MMA arms during the opening and closing actions were evaluated, as shown in Figure 3B(vi). A detailed theoretical investigation of the magnetic force, induced‐magnetic torque, and the arm open distance is provided in Supplementary Information 2. Supplementary Text. For a magnetic field intensity of 15 mT, the opening distance of a single arm was calculated to be 0.7 mm, resulting in a total arm span of 1.4 mm (Figure 3B(vi)). This estimation indicated that the MMA was capable of handling particles or micro‐objects with sizes ranging from 0.3 mm (default gap between the arms) to 1.4 mm. Meanwhile, an experimental validation was performed to ensure the accuracy and applicability of the theoretical predictions under practical conditions. The experimentally measured arm‐opening distance values were found to closely align with theoretical predictions, as shown in Figure 3B(vi, a), with an error margin of less than 5%. These results highlight the versatility of the presented MMA in executing diverse and precise motions, demonstrating its suitability for advanced microfluidic applications involving targeted manipulation and efficient handling of micro‐objects.

Figure 3
Analysis of the distinct motion strategies and corresponding trajectories of the MMA under various electromagnetic control schemes. A) Summary of the motion modes of the MMA, categorized into seven distinct movements. (I) Translational motion along the *x*‐axis, (II) translational motion along the *y*‐axis, (III) translational motion with an inclined trajectory, (IV) full rotational motion, (V) swinging motion, (VI) release, and (VII) grasping motions. The corresponding coil control strategies are illustrated for each mode, demonstrating the electromagnetic field configurations utilized to achieve the desired MMA movements. Further, the schematic representations indicate the initial and final positions of the MMA during each motion mode, with detailed descriptions of the applied control signals. The mathematical equations governing the *x*‐ and *y*‐axis displacements of the MMA during various motions are included for a comprehensive analysis. The blue color in the EMC diagrams represents the activated coils generating magnetic fields during a specific motion mode. B) The spatiotemporal analysis of the mentioned motions with their trajectories along the *x* and *y* axes. (i) Translational motion along the *x*‐axis was demonstrated, with the y‐position remaining constant over time as the MMA moved horizontally. Under the gradient magnetic field, the MMA reached its destination in a shorter time compared to the uniform magnetic field distribution. This behavior was attributed to the acceleration of the MMA as it approached the activated EMCs. (ii) Translational motion along the *y*‐axis was observed, where the *x*‐position remained unchanged while the *y*‐position increased linearly. Similar to horizontal navigation, a notable difference in the time required for vertical navigation was observed between the uniform and gradient magnetic field distributions. (iii) Translational motion along an inclined trajectory was shown, involving simultaneous changes in both *x* and *y* positions. Consistent with the horizontal and vertical navigation, a time difference in MMA movement along the inclined path was also observed between the uniform and gradient magnetic field distributions. (iv, a and b) Full rotational motion, validating the angular displacement against theoretical predictions for a complete rotation. (v, a and b) Swinging motion, where the periodic movements of MMA are demonstrated along the *x*‐axis. (vi, a and b) The opening and closing actions of the MMA for the release and grasping functions. The experimental results are plotted against theoretical predictions, showcasing minimal deviation and confirming the efficacy of the electromagnetic control strategies. The error bars represent a unit standard deviation in both directions, calculated from the results of three independent experiments.

**Figure:**
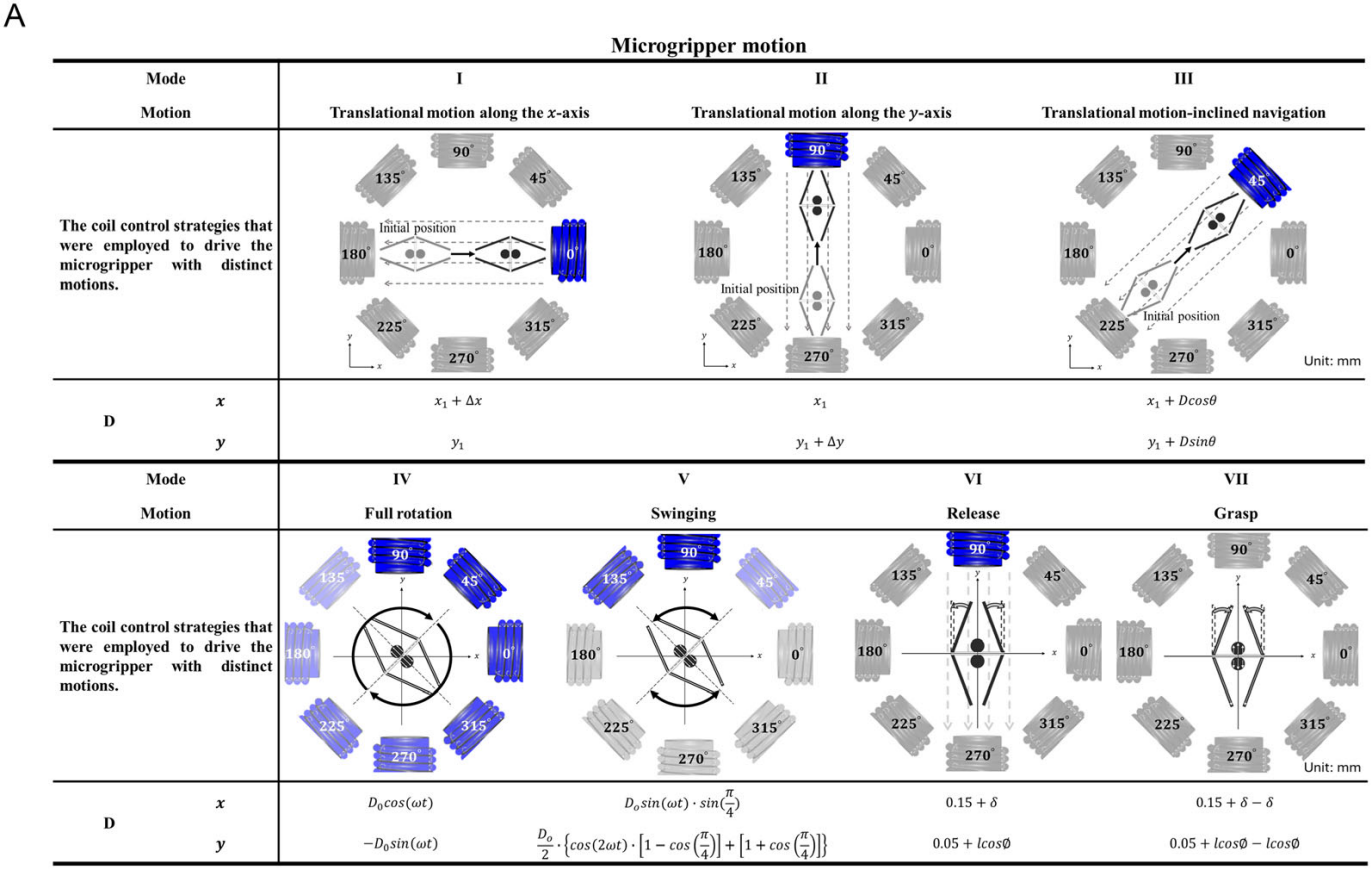

**Figure:**
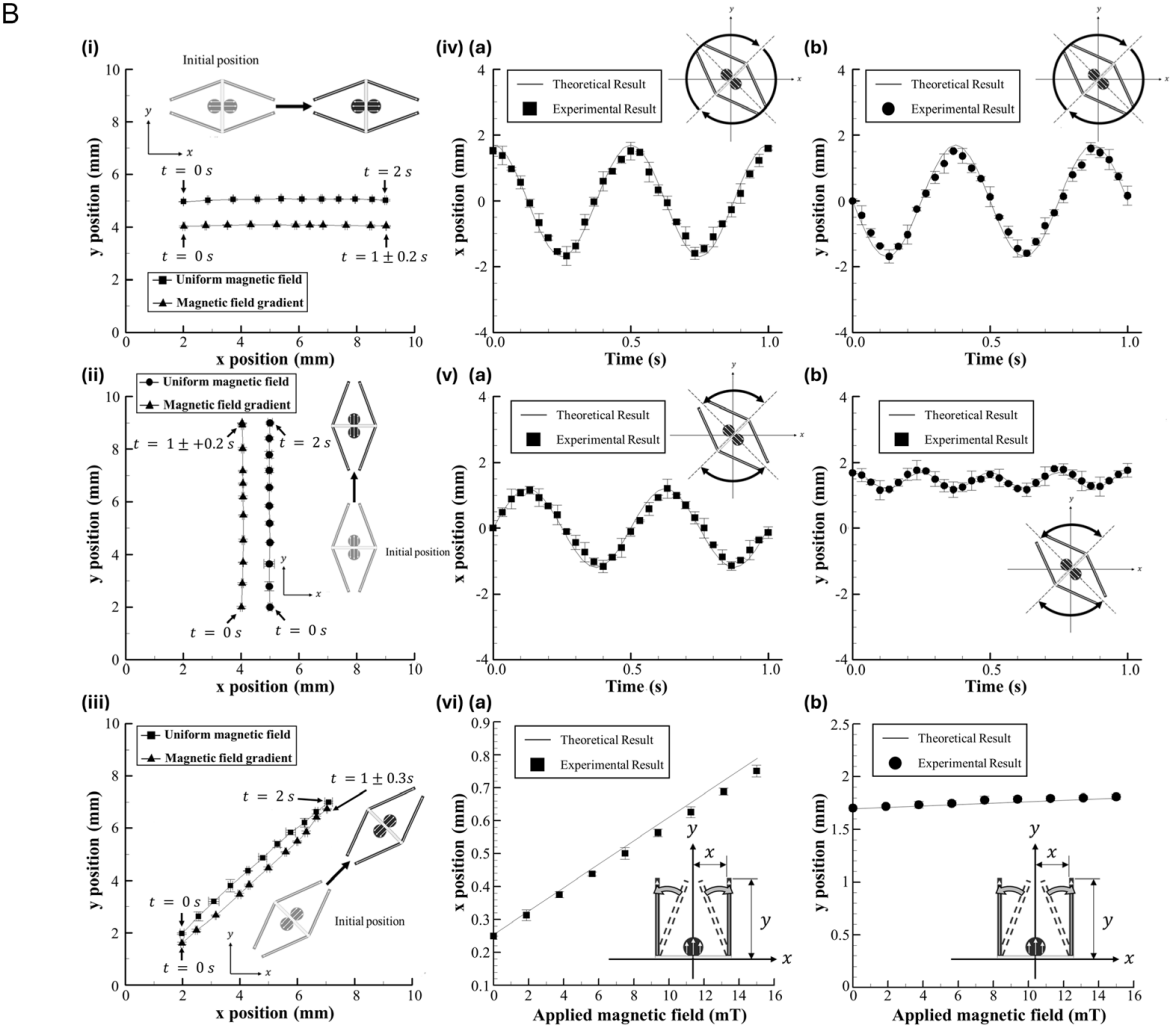

### 2.3 The Microgripper Function of MMA in Manipulating the Microparticles

The particle handling capability of micro‐robotic devices is crucial for precise manipulation in micro‐ and nanoscale environments, especially in biomedical and lab‐on‐a‐chip applications. This capability becomes crucial in multiparticle manipulation, as it enables tasks like cell sorting, microassembly, and high‐throughput testing.

**Figure 4:**
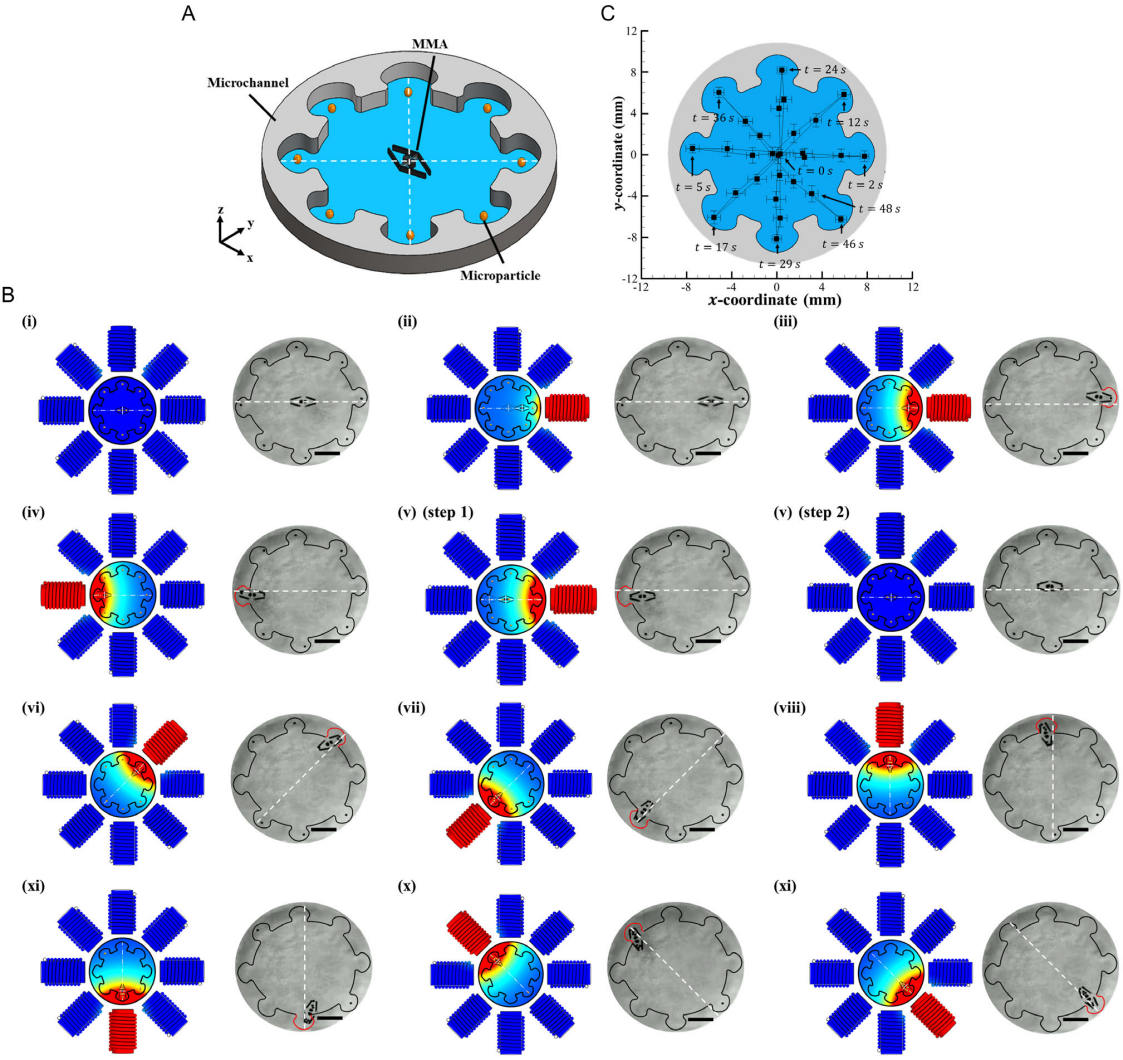
Schematic representation and experimental demonstration of the microparticle grasping function of the presented MMA. A) The customized microfluidic channel that was designed to demonstrate the MMA's ability to handle multiple microparticles within a single working cycle. The MMA, located at the center of the microchannel, was sequentially navigated to grasp eight strategically positioned microparticles. B) Detailed locomotion and grasping strategies of the MMA during the particle manipulation process. (i) Initial state of the MMA at the microchannel center. (ii) The MMA was navigated toward a particle located at 0° by activating the corresponding magnetic coil with a 0.5 A current, generating a magnetic field of 5 mT. (iii) The supplied current was increased to 1 A, enhancing the magnetic field to 15 mT, which facilitated the opening of the actuator arms and allowed the microparticle to be grasped. (iv–xi) Sequential activation of specific coils enabled the MMA to capture microparticles located at various angles (e.g., 180° and 45°) and load all eight particles between the actuator arms. The scale bar indicates 2 mm. C) Real‐time tracking results of the MMA, illustrating its path and successful capture of all eight particles within a 46 s timeframe. The error bars represent a unit standard deviation in both directions, calculated from the results of three independent experiments.

Moreover, the capability to handle multiple particles in a single working cycle increases efficiency and simplifies processes that demand the simultaneous manipulation of multiple entities, which is particularly advantageous in lab‐on‐a‐chip applications.^[^ ^32^ ^]^ In this work, the function of manipulating multiple microparticles in a single working cycle was demonstrated by employing the developed MMA. For demonstration purposes, a customized microfluidic channel was designed, with eight microparticles strategically positioned at distinct locations (shown in **Figure** **4**A and Video S1, Supporting Information). These microparticles were made up of PET (polyethylene terephthalate) material, each with a diameter of 300 μm and an approximate weight of 37 ng. The MMA was systematically controlled to grasp each of the eight particles from their respective positions. The corresponding locomotion strategies of the MMA, together with the coil activation approach, are shown in Figure 4B. Initially, the MMA was positioned at the center of the microchannel and navigated toward various particle locations by activating specific coils. For instance, to grasp the microparticle positioned at 0°, the coil at that angle was supplied with a 0.5 A current, generating a magnetic field of up to 5 mT. This value was observed to propel the MMA toward the target, as shown in Figure 4B(ii). Further, to facilitate the grasping function, the supplied current was then increased to 1 A, enhancing the field to 15 mT, which allowed the MMA arms to open (Figure 4B(iii)). Meanwhile, it can be observed that the grasped particle was in direct contact with the tip of the actuator arms only during the short interval of the grasping process. Once the microparticle was successfully grasped, it was then positioned within the region formed between a pair of actuator arms. This design feature was the key that enabled the device to collect multiple particles in a single cycle efficiently. In addition, this configuration can minimize the induced stress that could be developed during the grasping and transportation phases, as continuous contact between the particle and the actuator arm is reduced. This approach, thereby, can mitigate the risk of harm to delicate particles, such as viable cells. Together with the magnetic field's intensity, the MMA's proximity to the wall of the microchannel was observed to contribute significantly to the successful opening of the actuator arms. To capture additional particles, the MMA was navigated toward other positions inside the microchannel. For instance, to grasp a particle at 180°, the coil at that angle was activated (Figure 4B(iv)). Similarly, to access the corner particle at 45°, the MMA was first navigated to the channel center by activating the 0° coil and then released to maintain its central position. Subsequently, the 45° coil was activated, which propelled the MMA to capture the target (Figure 4B(v)). This process was repeated for each particle, allowing the MMA to load all eight microparticles (four on each side of the arms), as illustrated in Figure 4B(vi–xi). Further, the results of real‐time MMA tracking (Figure 4C) revealed that it successfully captured eight particles within a 46 s timeframe, achieving an average grasping time of 2–5 s per particle positioned 4–8 mm apart within the microchannel. This timing was observed to be superior to that of their alternatives, such as a flexible magnetic actuator equipped with six gripping arms operated at 10–30 mT, which was reported to necessitate 36 s for single‐object manipulation per working cycle.^[^ ^32^ ^]^ Further, unlike the manipulation of uniformly sized particles, the MMA was employed to grasp particles of varying sizes, ranging from 300 to 900 μm. A detailed illustration of this process is provided in Figure S4, Supporting Information. However, while the presented MMA's design enabled sophisticated multiparticle grasping, selective release could pose a limitation. Since particles were secured between the actuator arms throughout the operation, releasing specific particles selectively might be challenging.

This limitation suggests potential for future design enhancements to support selective, efficient particle release while maintaining the precise handling observed during multiparticle grasping. Nevertheless, the presented MMA showcased notable advancements in manipulating the particles, including its enhanced adaptability in handling multiple objects and improved versatility, which was provided by independent arms control.

### 2.4 Microassembly Task Executed by the MMA

**Figure 5:**
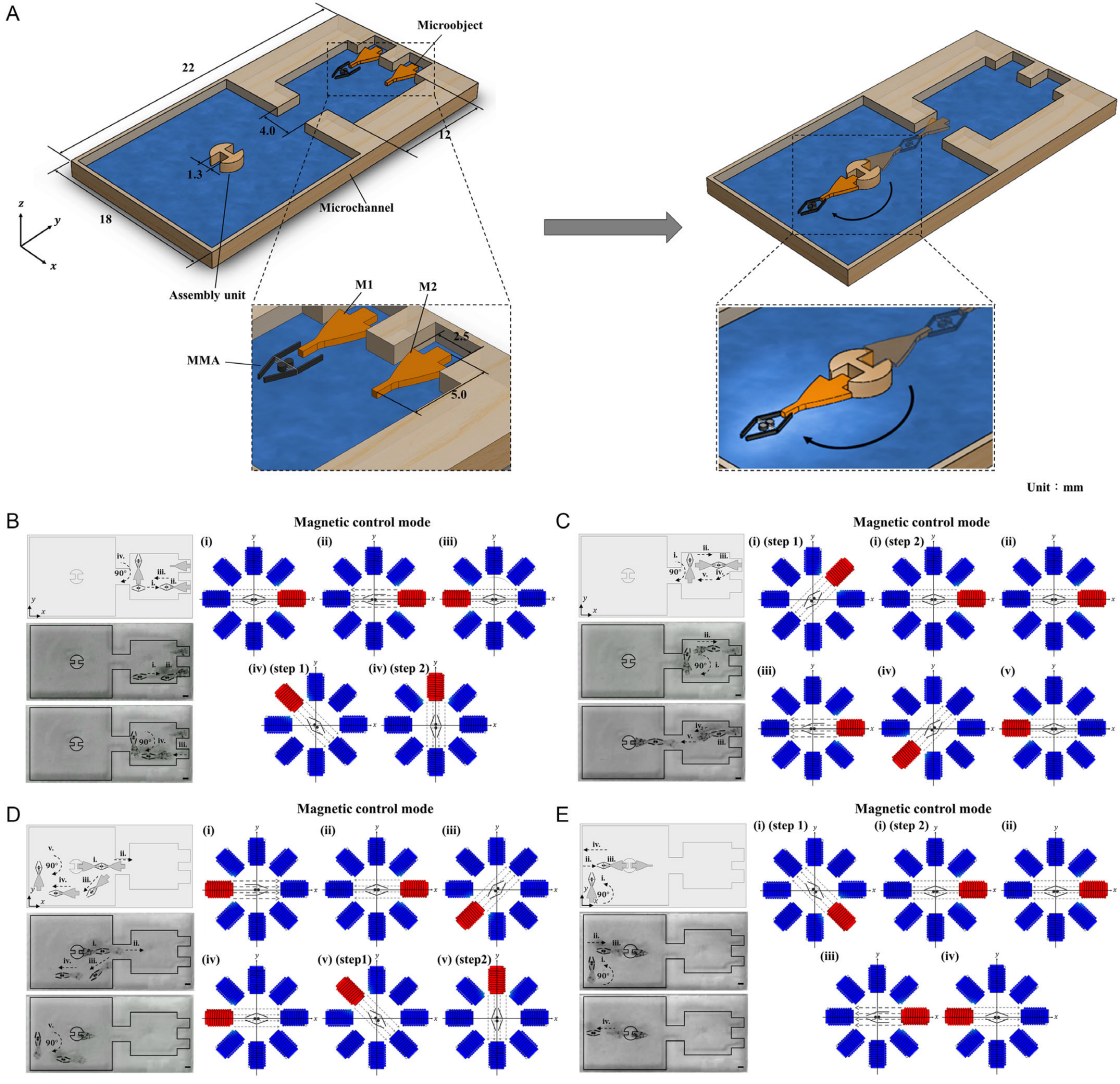
A microassembly task performed by the presented MMA within a microfluidic environment. A) The illustration of the microfluidic channel that was designed for the sequential assembly of two micro‐objects (M1 and M2) at a designated assembly site. The inset shows a magnified view of the MMA handling a micro‐object within the fluid environment. The image on the right side shows the assembled state of M1 and M2 at their designated assembly site. B) The illustration of the sequential microassembly process through the captured optical images and together correlated with the activation sequence of EMCs. (i) The MMA was navigated toward M1 by activating the 0° EMC with a 0.5 A current. (ii) Upon reaching M1, a current of 1.5 A was applied to generate a magnetic flux density of 15 mT, facilitating the opening of the MMA's arms and grasping of M1. (iii) The MMA was subsequently navigated back to its initial position to prepare for the capture of M2 by activating the 180° EMC. (iv) To align with M2's position, the MMA was rotated and navigated along the vertical axis. C) Loading of M2 was achieved by opening the arms with a 1.5 A current and navigating the MMA along the horizontal axis by activating the appropriate EMCs. (v) Both M1 and M2 were transported to the assembly station, where the assembly process was initiated. D) The MMA was manipulated to release M1 and M2 sequentially into their designated slots within the assembly station. (i) M1 was positioned and released by activating the 180° EMC, increasing the current to 1.5 A to open the arms and unload M1. (ii–iv) The MMA was subsequently repositioned and rotated to release M2 into the corresponding assembly slot. E) Final stages of the assembly process, highlighting the precise placement of M1 and M2 at the designated assembly site. The scale bar represents 5 mm.

Microassembly within liquid microenvironments is essential for various microfluidic applications, including the development of lab‐on‐a‐chip devices, biosensors, and microelectromechanical systems (MEMS). This process enables precise manipulation and arrangement of microscale components, facilitating the construction of functional microstructures and improving the integration of microfluidic systems with other technologies. Numerous methodologies have been proposed to facilitate the microassembly task, including the actuators driven by acoustic fields,^[^ ^8^ ^]^ shape memory alloys (SMAs),^[^ ^7^ ^]^ electrostatic forces,^[^ ^33^ ^]^ and magnetic fields.^[^ ^25^, ^34^ ^]^ For instance, the acoustic field‐driven approach utilized ultrasonic waves to manipulate a single micro‐object per working cycle by employing the two fixed ultrasonic phased array (UPA) systems.^[^ ^8^ ^]^ In this configuration, the assembly of micro‐objects was executed by the ultrasonic wave modulation controlled by individual transducers within the array. In the case of the SMA‐based actuator,^[^ ^7^ ^]^ the electric current between 0.8 and 1.6 A was supplied to actuate a pair of gripper‐based arms for loading a single cargo with an average dimension of 2.07 mm. As a strategy for enhancing the assembly process, a dual manipulator‐based actuator was introduced to assemble a single cargo.^[^ ^35^ ^]^ By employing this approach, a rectangular silicon cargo was manipulated back and forth by dual manipulators, successfully assembling it in the predefined target zone with an 83% success rate. However, while push‐and‐pull methods were primarily emphasized for assembly, techniques involving the grasping, holding, and releasing of micro‐objects have been shown to significantly enhance the efficiency of the microassembly process, enabling more advanced and intricate assemblies.^[^ ^36^ ^]^ To realize the latter approach, a gripper‐based magnetic actuator was introduced with a pair of gripper arms embedded with permanent magnets.^[^ ^25^ ^]^ Subsequently, the gripping action was established as the result of the magnetic torques induced on the arms upon being subjected to the external magnetic field. Then, a triangular‐shaped assembly of three microspheres, each with a diameter of 200 μm, was demonstrated by employing multiple gripper agents. These grippers were maneuvered through external magnetic fields with strengths of 5 mT to facilitate precise orientation and navigation. Despite these advancements, the discussed current approaches are constrained by limited handling capacity, as many microactuators are designed to handle only a single object per operational cycle, in addition to the requirement for precise motion control at microscale resolutions. Moreover, the ability to navigate complex geometries or reach constrained regions within microchannels remained inadequate, thus limiting their utility for intricate assembly operations. To address these challenges, this study proposed an alternative approach by employing the presented MMA capable of performing high‐throughput sequential microassembly tasks. The proposed actuator was designed to handle two objects per working cycle, facilitating the sequential loading, transportation, and unloading of cargo at a designated assembly unit. In addition, the proposed MMA was equipped with dedicated mobility components, which facilitated superior navigational control. These features allowed the actuator to reach complex and confined areas within the microchannel, ensuring the successful loading and unloading of cargo even in narrow environments. For demonstration purposes, two distinct yet identical micro‐objects (M1 and M2) were sequentially captured from their respective locations, transported, and then sequentially released to assemble them within the designated assembly region, as depicted in **Figure** **5**A and Video S2, Supporting Information. During this process, the pair of arms in MMA was controlled to open 1.4 mm (each arm opening to 0.7 mm) under an externally applied magnetic field of 15 mT (a detailed analysis is provided in Figure 2C and 3B(vi)). Further, through theoretical analysis, it was calculated that each arm exerted the gripping force (reaction force) of 20 μN under the same magnetic field intensity (a detailed discussion is provided in the Supplementary Information, 2. Supplementary Text). This calculated value aligned closely with previous experimental observations, which measured the grasping force of microgripper‐based MMAs within a range of 20 ± 5.5 μN.^[^ ^37^ ^]^ The placement of the assembled micro‐objects in the assembly station is depicted on the right side of Figure 5A. Further, a detailed illustration of the sequential loading, transportation, and assembly processes of the micro‐objects, captured as optical images during the experiment, is shown in Figure 5B,E. The corresponding activation sequence of the EMCs, responsible for MMA's arm movement and navigation, is presented adjacent to each of these subfigures. For instance, Figure 5B illustrates the sequence of operations commencing with the loading of micro‐object M1 and proceeding to a step prior to the capture of micro‐object M2. This sequence was further delineated into four discrete stages, designated as (i) through (iv). Stage (i) represents the navigation of the MMA from its initial position to the location of M1 for loading. This motion was achieved by applying a 0.5 A current to the EMC coil positioned at 0°. Once the MMA was navigated to the vicinity of M1, its arms were opened to facilitate the loading process. To perform this function, the supplied current was increased to 1.5 A, generating a magnetic flux density of 15 mT (Figure 5B(ii)). After successfully securing M1, the MMA was navigated back to its initial position to prepare for loading M2. This backward motion was executed by activating the EMC positioned at 180°, as shown in Figure 5B(iii). Since the position of M2 within the microchannel was vertically above M1 (a higher *y*‐axis value), the MMA was rotated 90° from its current orientation to align along the vertical axis (Figure 5B(iv)). This adjustment allowed the MMA to navigate to the horizontal axis corresponding to the position of M2. Once aligned with the horizontal axis, the MMA was rotated back to its initial orientation and navigated toward M2 with its arms opened for loading, as depicted in Figure 5C. This motion was executed by supplying the current of 1.5 A to the EMC positioned at 0° (Figure 5C(ii)). After loading M2, the MMA was navigated back to its initial position with both arms carrying M1 and M2 to prepare for their assembly in the assembly station (Figure 5C(v)). Then, the sequential assembly of the loaded micro‐objects was initiated by positioning the MMA near the assembly station (Figure 5D). First, M1 was assembled by navigating the MMA toward the designated assembly slot by employing the EMC positioned at 180° with a current of 0.5 A. To release M1 precisely into the assembly slot, the supplied current was increased to 1.5 A, causing the arms to open and unload M1, as shown in Figure 5D(i). Following the assembly of M1, the MMA was manipulated to assemble M2 on the opposite side of the assembly site. This process involved sequential translation and rotation of the MMA, as depicted in Figure 5D(iii),(iv), to align it near the designated slot for M2 in the assembly station. The MMA was then navigated near the assembly site of M2 by supplying a current of 0.5 A to the EMC positioned at 0°. Finally, M2 was released into its slot by increasing the current to 1.5 A, enabling the arms to open and unload precisely, as illustrated in Figure 5E(i),(iv). It is important to note that the mobility components attached to either side of the MMA played a crucial role in influencing its movement dynamics, ensuring stable navigation across different cross‐sectional regions of the microchannel (a detailed analysis of the mobility components’ dynamics is provided in Section 2.1). By incorporating these design improvements, the proposed MMA demonstrated enhanced capability in performing microassembly tasks within a fluid environment. This approach demonstrated high throughput while addressing the limitations of conventional methodologies. This study's findings highlight the proposed MMA's potential to advance microassembly processes, providing promising solutions for applications that require precise and efficient manipulation of microscale components within microfluidic systems.

### 2.5 The Micromixing and Fluid Flow Conveyance Tasks Executed by the MMA

**Figure 6:**
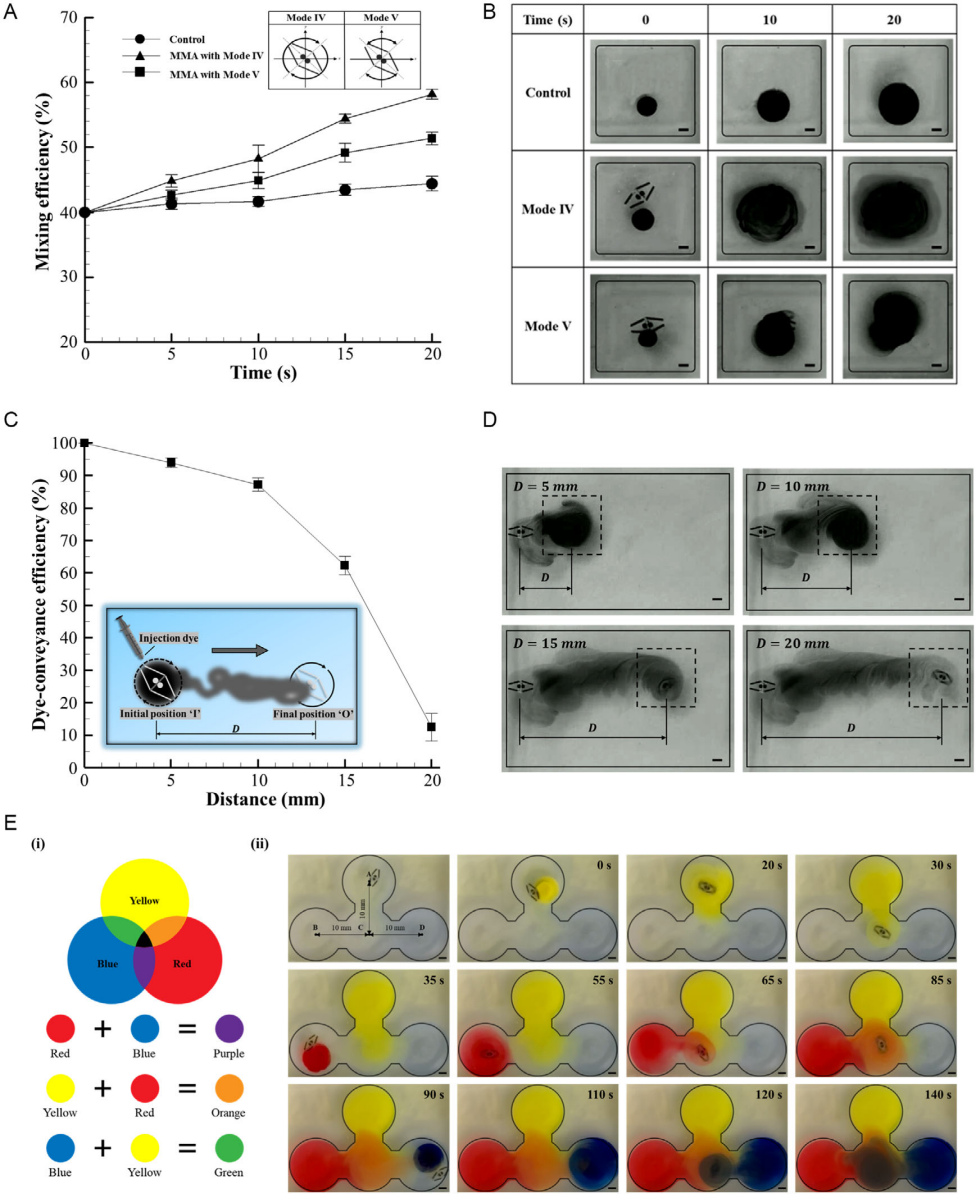
Experimental demonstration of micromixing and fluid flow conveyance tasks executed by MMA. A) The micromixing performance of the MMA was evaluated by injecting methylene blue dye into water within a microchannel. The MMA was controlled to execute two distinct stationary rotational motions, including Mode IV (complete or full rotation) and Mode V (swinging motion). B) Optical images captured at different time intervals (0, 10, and 20 s) demonstrated the superior mixing efficiency of Mode IV compared to Mode V. Mode IV was observed to facilitate a uniform and global dispersion of the dye within the channel, achieving a mixing efficiency of 61% after 20 s, while Mode V resulted in localized mixing with an efficiency of 49%. C) The fluid conveyance capability of the MMA was assessed by transporting methylene blue dye from an initial position (I) to a target location (O) within the microchannel. The conveyance was achieved by actuating the MMA with combined rotational and translational motions. D) Optical images illustrate the dye conveyance over varying navigational distances (5, 10, 15, and 20 mm), showing a decrease in conveyance efficiency with increasing distance. E) The demonstration of combined fluid conveyance and mixing performance within a specially designed microchannel. (i) The standard color topology illustrates that the intersection of the primary colors yellow, red, and blue results in the formation of black. (ii) The fluid conveyance experiment was performed inside the microchannel by transporting yellow, red, and blue dyes from zones A, B, and D to a common target zone C. Then, the MMA was subsequently actuated with rotational motion at zone C to blend the dyes. The optical images captured during the operation depicted the emergence of a black color, confirming efficient mixing. The error bars represent a unit standard deviation in both directions, calculated from the results of three independent experiments.

An efficient fluid mixing is essential in numerous microfluidic applications, including drug delivery systems, biochemical assays for biomarker detection, lab‐on‐chip platforms, and point‐of‐care diagnostic devices. However, the presence of laminar flow within the microchannel can impede the mixing process, as it relies solely on slow molecular diffusion, thereby diminishing mixing efficiency. To overcome this limitation, various devices, including active micromixers,^[^ ^38^, ^39^, ^40^ ^]^ have been introduced where the slow diffusion phenomenon was enhanced by inducing the convective effects. For example, a dielectrophoretic micromixer was proposed to induce polarization in the fluid or suspended particles upon being subjected to an external electric field.^[^ ^41^ ^]^ As a result, the localized mixing zones were created by leveraging variations in the dielectric properties of the fluid or particles, thereby improving the mixing efficiency. Similarly, a micromixer employing a magnetic field was designed to generate dynamic flow patterns within the microchannel to enhance the rate of fluid mixing.^[^ ^42^ ^]^ However, these strategies are constrained by their reliance on specific fluid properties, such as electrical conductivity or magnetic susceptibility, limiting their applicability to a broader range of fluids. To address these challenges, this study proposed a physical agitation‐based strategy utilizing the presented MMA. Further, this approach of mechanical agitation generated through the distinct rotational modes of the MMA was found to significantly enhance the mixing efficiency. For the purpose of demonstration, the fluid mixing ability of the MMA was experimentally tested within a microchannel by injecting a few drops of methylene blue dye into water. A comprehensive description of the experimental procedure for the mixing task is provided in the “Mixing Performance” section of the Experimental Section. To induce the mixing between the methylene dye and water, the MMA was magnetically controlled to execute distinct modes of rotational motion, such as Mode IV and Mode V (a detailed discussion on the dynamics of these rotational modes is provided in Section 2.2). In the demonstration, the MMA was placed at the center of the microchannel, where a dye was injected and controlled to perform distinct stationary rotational motions. This approach was based on previous studies, which showed that positioning microswimmers along or within fluid flow paths effectively facilitates the stretching and folding of fluid layers, significantly improving mixing performance.^[^ ^43^ ^]^ As illustrated in **Figure** **6**A, Mode IV was observed to achieve significantly higher mixing efficiency compared to Mode V over the same duration. For instance, after 20 s of mixing, Mode V can be seen to reach the mixing efficiency of 49%, whereas Mode IV attained 61%. It should also be mentioned that the prolonged mixing durations could further improve the efficiency of both modes. The optical images that were captured during the mixing experiments are shown in Figure 6B. From this figure, the critical fluid dynamics associated with each mode of MMA rotation can be interpreted. For example, the full rotation motion of MMA (Mode IV) was observed to induce a uniform dispersion (or diffusion) of fluid (dye) inside the microchannel. Consequently, a more extensive distribution (global) of the dye within the microchannel was noted, which resulted in a better mixing efficiency when quantified over the entire channel area. In contrast to this behavior, the swinging rotational motion of MMA (Mode V) was observed to induce a pulsating fluid flow across the confined region inside the microchannel. As a result, the more localized fluid mixing was noted that limited the extensive diffusion, leading to a lower mixing efficiency compared to Mode IV. Overall, this study demonstrated that the mechanical agitation introduced by the MMA, through its distinct rotational dynamics, offers a robust and adaptable solution for enhancing fluid mixing while overcoming the limitations of other mixing techniques and their dependence on fluid properties.

In addition to microfluidic mixing, the fluid transportation function (the fluid flow conveyance) was demonstrated by employing the presented MMA. Similar to micromixing, the fluid flow conveyance within the microchannel is also a critical process in all microfluidics applications. Conventionally, pumps,^[^ ^44^ ^]^ pressure‐driven mechanisms,^[^ ^45^ ^]^ surface‐driven passive pumping techniques,^[^ ^46^ ^]^ valves,^[^ ^47^ ^]^ and nozzles^[^ ^48^ ^]^ have been integrated within microfluidic systems to facilitate the fluid handling and transport function through the flow conveyance approach. However, these methods required the incorporation of tubing and interconnections to interface the microfluidic device with external pumps or pressure sources. The reliance on external components may increase the overall fluid sample volume and dead volume while reducing the portability of the microfluidic device. As an alternative approach, a carpet thread,^[^ ^49^ ^]^ chosen for its absorbent properties and capillary potential, was employed as a medium for fluid conveyance that enabled fluid flow transport of volume between 2 and 10 μL (in 60 s) from different sections inside the microchannel. However, the effectiveness of this method was constrained by challenges such as the material properties of the tissue paper, the evaporative and non‐evaporative characteristics of the working fluid, and the requirement to maintain the thread in a saturated state for consistent fluid conveyance. These limitations posed significant obstacles to its broader applicability in various microfluidic systems. This part of the presented study highlighted that the proposed MMA could serve as an efficient alternative for fluid conveyance within microchannels, eliminating the dependency on valves, pumps, or nozzles typically integrated into microfluidic devices. Here, the conveyance process was achieved by actuating the MMA with a combination of rotational and translational motions within the microchannel, as illustrated in Figure 6C. For demonstration purposes, 15 μL of methylene blue dye was introduced into the microchannel at a designated position labeled “I”, with the MMA initially positioned nearby. The combined motions of the MMA were then activated to transport the dye to a target location labeled “O”. The dye conveyance was facilitated through the dragging action induced by the no‐slip condition between the dye and the MMA. The operation was conducted over a duration of 30 s, during which the MMA transported the dye from the initial position “I” to the final position “O”. The conveyance capability of the MMA was further evaluated over varying navigational distances within the microchannel, specifically 5, 10, 15, and 20 mm. A decrease in conveyance efficiency (an expression to calculate the dye‐conveyance efficiency is provided in Supplementary Information, 2. Supplementary Text, 4. Dye‐conveyance efficiency (DCE)) was observed as the MMA's navigational distance increased. For instance, a conveyance efficiency of 90% was achieved at 5 mm, which reduced significantly to 59% at 15 mm and further declined at 20 mm. To substantiate these findings, the volume of dye conveyed was experimentally measured for different navigational distances of MMA. The volume of conveyed dye for 5, 10, 15, and 20 mm of MMA's navigational distances were determined to be 12, 10, 6, and 3 μL within the 30 s operational period. These variations in conveyance volume with increasing navigational distance were corroborated by optical images captured during the experiments, as shown in Figure 6D. Though fluid dragging was the primary mechanism driving the conveyance, simultaneous dye diffusion into the surrounding medium was also observed. As the MMA's travel distance increased, this diffusion became more significant, reducing conveyance efficiency and volume. To mitigate this, the conveyance task could be performed in a confined tubular structure rather than an open microchannel, as employed in this study. Nevertheless, based on the considered experimental setup and MMA dimensions, an optimal navigational distance was identified to be 10 mm, achieving the dye conveyance efficiency of 85% within 30 s. To validate this finding, additional experiments were conducted in which the MMA was actuated to convey three distinct colored dyes, such as yellow, blue, and red, within the microchannel, as shown in Video S3, Supporting Information. Following this conveyance, the MMA was further controlled with the rotational motion to facilitate mixing. In particular, the different colored dyes were initially transported from zones A, B, and D to a common target zone C. Subsequently, the MMA's rotational motion was executed at zone C to blend the dyes, producing a black color indicative of efficient mixing. To illustrate the mixing outcome, a standard color topology of the selected color dyes is provided in Figure 6E(i), where the intersection of yellow, red, and blue yields black. The optical images captured during the experiments, as shown in Figure 6E(ii), depict the sequential execution of the conveyance and mixing processes. After 140 s operation (encompassing both conveyance and mixing steps), the emergence of the black color was observed, confirming the MMA's capability to effectively manipulate fluid flow within the microchannel by demonstrating its suitability for both robust conveyance and efficient mixing tasks.

## 3 Conclusion

In this study, the small‐scale magnetic actuator was developed to perform multiple functions, including particle manipulation, micro‐object assembly, and fluid flow control within the microfluidic environment. By integrating the mobility components into the MMA's architecture, navigation precision was enhanced, enabling the actuator to execute complex tasks with high efficiency, even in confined environments. The successful manipulation of particles and micro‐objects at high throughput, coupled with the control of fluid flow for micromixing and conveyance, demonstrated the potential synergistic advantages of the presented MMA. Further, the findings revealed that the presented system can serve as a solid foundation for the future development of high‐throughput, versatile, small‐scale actuators that could significantly enhance the performance and efficiency of microfluidic processes.

## 4 Experimental Section

4.1

4.1.1

##### Design and Fabrication of MMA

The MMA was fabricated with both magnetic and nonmagnetic sections. The magnetic regions were composed of a composite material, which included neodymium–iron–boron (NdFeB) magnetic particles embedded in PDMS. In contrast, the nonmagnetic sections were made from pure PDMS. PDMS was selected as the base material due to its advantageous properties, such as biocompatibility, flexibility, elasticity, optical transparency, ease of fabrication, and cost‐effectiveness. These attributes have made PDMS a widely employed material in microfluidic research and its applications.^[^ ^50^, ^51^, ^52^, ^53^, ^54^, ^55^, ^56^, ^57^, ^58^, ^59^, ^60^ ^]^ In this study, PDMS was utilized to form the structural framework of the MMA. The magnetic sections, including the arms (responsible for grasping and releasing functions) and the eyes (for motion control), were embedded with a mixture of NdFeB particles and PDMS, while the remaining sections were composed of pure PDMS. The PDMS material was prepared by combining pure PDMS and curing agents (Sylgard 184, Dow Corning Corp., Midland, MI, USA) in a weight ratio of 10:1.^[^ ^61^ ^]^ The magnetic sections were fabricated by mixing 5 μm NdFeB magnetic particles (MQP‐15‐7, Magnequench International, Inc., Singapore) with PDMS in a ratio of 4:1.^[^ ^62^ ^]^ The fabrication of the MMA involved seven key steps, which are detailed in Figure S1, Supporting Information.

##### The Magnetic Drive System

The MMA was controlled externally by employing a custom‐built system comprising eight electromagnetic coils (EMCs) arranged at 45° intervals around the device. Each EMC was constructed by winding 1200 turns of copper wire around a carbon steel bar measuring 15 mm in length and 2 mm in width. A data acquisition device (NI DAQ‐9174, National Instruments, Austin, TX) was employed to regulate the motions of the MMA. These modules were interfaced with the EMCs and an external power source (GPR‐3510HD DC Power Supply, Instek, Taiwan) to generate the magnetic fields necessary for motion control. The magnetic field required for MMA actuation was produced by the aforementioned system, and its operations were managed by employing a LabVIEW‐based graphical user interface developed by National Instruments. The MMA's movements were documented using a charge‐coupled device (CCD) camera (WAT‐902H ULTIMATE, WATEC, Japan), which was equipped with a microlens (AF Micro‐NIKKOR 60 mm f/2.8D, Nikon, Japan). The camera operated at a frame rate of 30 frames per second and effectively recorded the MMA's motions, providing detailed visual data for further analysis and observation.

##### Microparticles and Objects’ Manipulation

In this study, microparticles and 2D planar micro‐objects were employed to demonstrate the MMA's capabilities in gripping and assembly processes. A camera system was employed to monitor and record the motion of these objects during experimental trials. The recorded image sequences were analyzed using ImageJ, an open‐source software tool. The image sequences were imported into ImageJ, where manual marking of the particle positions was conducted frame by frame to generate motion trajectories. Once the trajectories were established, the instantaneous velocities of the particles were calculated by determining the distance traveled over specified time intervals. A calibration process was conducted to establish a correlation between the pixel count in the images and the corresponding physical distances, ensuring measurement accuracy. The resulting data were then exported for further analysis, and all required calculations were performed. This included the motion dynamics of MMA, as well as the gripping and assembly performance of micro‐objects.

##### Mixing Performance

In this experiment, the MMA was initially positioned inside a microchannel that was partially filled with a 40% aqueous glycerol solution. Methylene blue dye, with a concentration of 100% by weight, was subsequently introduced at specific locations within the microchannel using a pipette. Following this, the MMA was actuated using electromagnetic coils (EMCs) to execute distinct motion patterns, including Modes IV (complete or full rotation) and V (swinging motion). The entire mixing operation was documented using a camera system, and the resulting images were analyzed using the open‐source software, ImageJ. This software was utilized to process the recorded images and extract critical parameters required for determining the mixing efficiency.^[^ ^63^, ^64^, ^65^, ^66^ ^]^

(1) $$\text{Mixing efficiency }(\%)=\left(1-\frac{1}{\bar{m}}\sqrt{\frac{\sum_{i}^{n}{\left(m_{i}-\bar{m}\right)}^{2}}{n}}\right)\times 100$$

where *m*_*i*_ represents the intensity of individual pixels, $\bar{m}$ is the mean pixel intensity, and *n* is the total number of pixels.

##### Statistical Analysis

All experiments were conducted in triplicate (*n* = 3) to ensure reproducibility and reliability of the results. Data are presented as mean ± standard deviation (SD), indicating the variability within the experimental outcomes. The standard deviation was calculated to assess the consistency of the measurements across the three experimental trials.

## Conflict of Interest

The authors declare no conflict of interest.

## Author Contributions

**Dineshkumar Loganathan**: writing—original draft (lead). **Chia‐Hsin Cheng**: data curation (lead); formal analysis (lead). **Po‐Wei Wei**: data curation (supporting); formal analysis (supporting). **Chia‐Yuan Chen**: conceptualization (lead); funding acquisition (lead); writing—review and editing (lead). **Dineshkumar Loganathan** and **Chia‐Hsin Cheng** contributed equally to this work.

## Supporting information

Supplementary Material

## Data Availability

The data that support the findings of this study are available from the corresponding author upon reasonable request.
