# Supplementary material for: In‐Liquid Micromanipulation via a Magnetic Microactuator for Multitasking
Source: Small Sci. 2025 Mar 30;5(7):2500010. doi: 10.1002/smsc.202500010 (PMC12257887; doi:10.1002/smsc.202500010)
Supplement: Supplementary file 1 — Supplementary Material [file SMSC-5-2500010-s001.zip › smsc202500010-sup-0001-SuppData-S1.pdf]

# Supporting Information

## In-Liquid Micromanipulation via a Magnetic Microactuator for Multitasking

*Dineshkumar Loganathan*[¥]*, Chia-Hsin Cheng*[¥]*, Po-Wei Wei, and Chia-Yuan Chen**

**This supporting information file includes:**

**1. Supplementary Figures S1 to S5**

**Figure S1**. Schematic illustration of the presented magnetic microactuator (MMA) fabrication process.

**Figure S2.** The arm opening distance of MMA.

**Figure S3**. Simulated magnetic field distribution for achieving the translational motion of MMA.

**Figure S4.** The versatility of the presented MMA in grasping different sizes of microparticles.

**Figure S5**. The current-phase difference method to achieve the rotational motion of MMA.

**2. Supplementary Text**

1. The expressions to calculate the MMA arm's magnetic force, torque, stiffness, displacement, and grasping force

2. Magnetic field-induced force, torque, and displacement analysis of the magnetic arm and the associated grasping force on the grasped particles

3. Investigation of the MMA's arm performance based on material selection

4. Dye-conveyance efficiency (DCE)

**3. Legend for Supplementary Videos S1 to S3**

**Video S1**. Sequential grasping of eight microparticles by MMA.

**Video S2**. The microassembly task executed by MMA.

**Video S3**. The flow conveyance and micromixing tasks executed by MMA.

## 1. Supplementary Figures S1 to S5

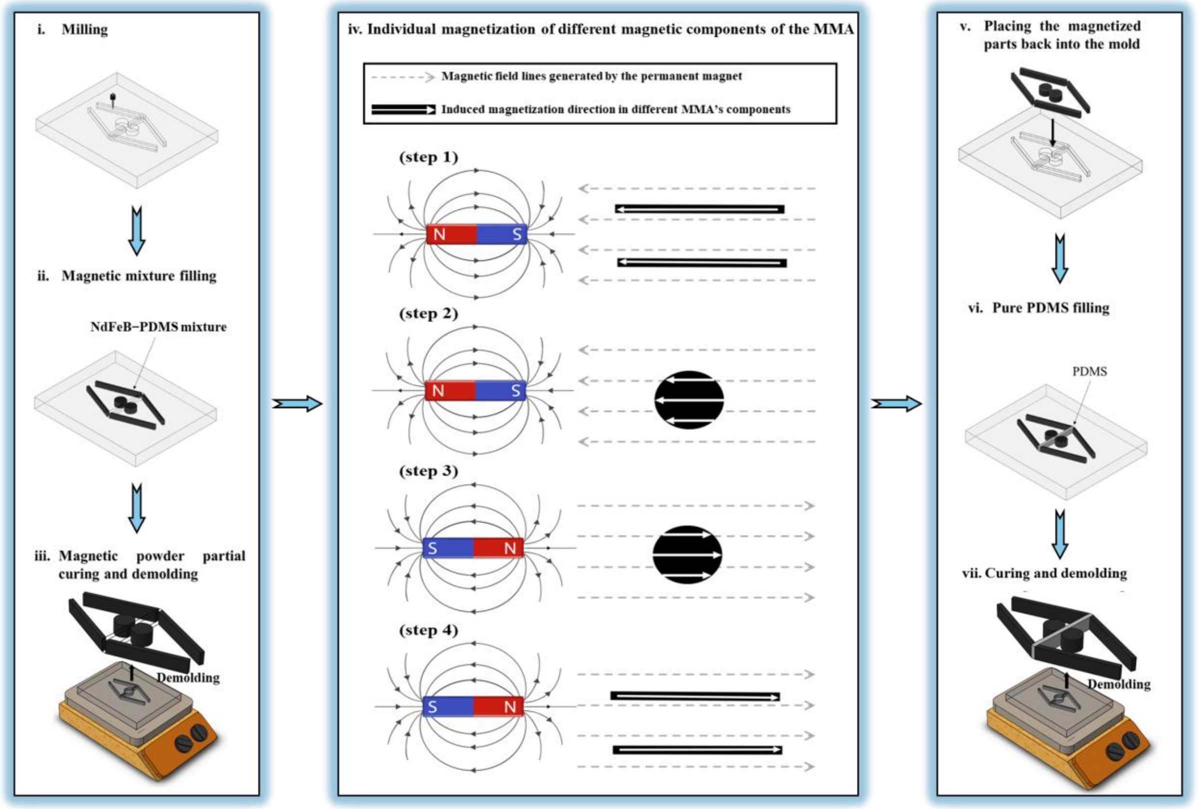


**Figure S1**. **Schematic representation of the fabrication process for the presented MMA.** The fabrication process comprised eight key steps to create the final structure of MMA. **Step (i):** The initial design of the MMA was developed using commercial computer-aided design software, followed by engraving the design onto an acrylic substrate through CNC machining to serve as the mold. **Step (ii):** A mixture of polydimethylsiloxane (PDMS) and NdFeB magnetic particles was carefully introduced into the mold designed for the MMA structure. **Step (iii):** The filled mold was subjected to a thermal curing process at 85°C for 2 hours to solidify the mixture. Subsequently, the cured magnetic components, including the arms and the central "eye" structure of the MMA, were removed from the mold for magnetization. **Step (iv):** These components were magnetized individually by employing a permanent magnet to ensure precise magnetic properties. **Step (v):** The magnetized components were repositioned within the mold. **Step (vi):** Pure PDMS was injected into the mold to bond the components together and form the complete MMA structure. **Step (vii):**The mold underwent a secondary curing process to ensure full integration of the components. After curing, the final MMA was demolded. The fabricated MMA had

dimensions of 3.39 mm in length, 1.72 mm in width, and 0.3 mm in thickness, ensuring its suitability for micro-manipulation tasks.

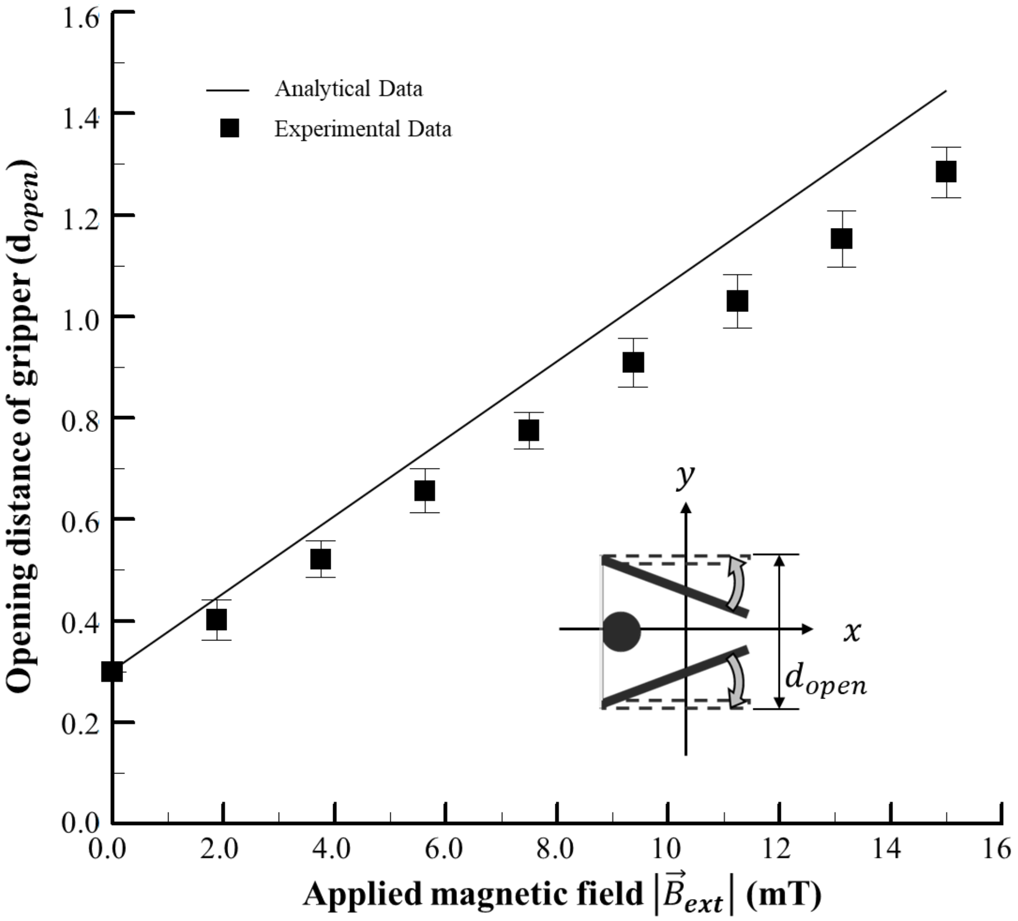


**Figure S2. The arm opening distance of MMA**. The application of an external magnetic field was observed to induce a magnetic torque in the arms of the MMA, causing them to deflect and perform coordinated motions resembling opening and closing actions, facilitating the release and grasping of particles or objects. The arm opening distance can be seen to increase proportionally with the strength of the applied magnetic field. As the value of magnetic field strength increased, the arms exhibited greater deflection (or opening). For example, at a magnetic field strength of 2 mT, an opening distance of 0.4 mm was observed, which progressively increased to a maximum of 1.4 mm at 15 mT. Further, it should be noted that a default arm opening distance of 0.3 mm was incorporated into the design to ensure a minimal gap between the arms, even in the inactive state. With this demonstration, it can be observed that the presented MMA was capable of grasping particles or objects with sizes in the range of 0.3 mm < $d_p$ < 1.4 mm, where $d_p$ represents the diameter of microparticles. The error bars represent a unit standard deviation in both directions, calculated from the results of three independent experiments.

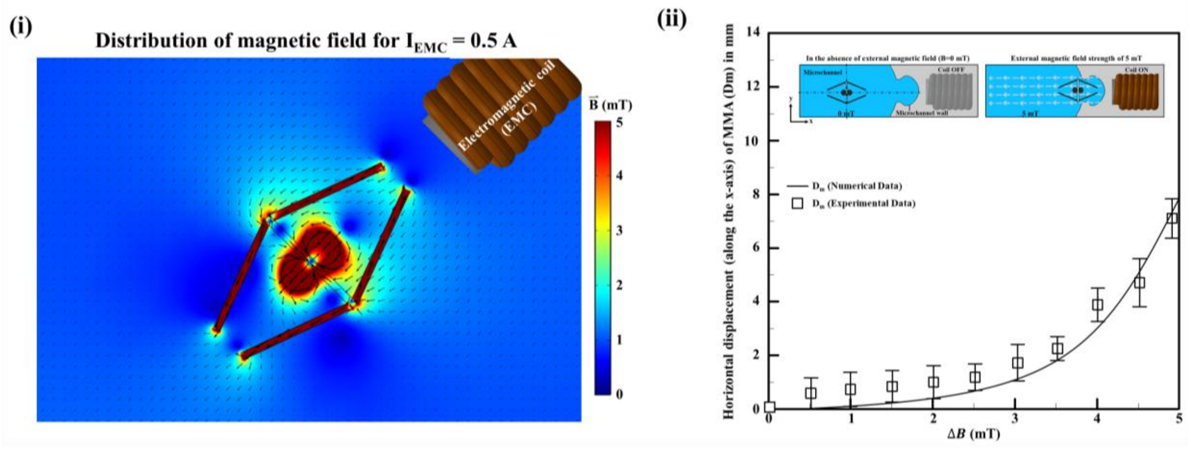


**Figure S3. Magnetic field distribution and displacement analysis of the MMA under varying magnetic field strengths**. (i) The numerical simulation of the magnetic field distribution around the MMA for a current of $I_{EMC}$=0.5 A applied to the EMC. The simulation displayed the spatial distribution of the magnetic field intensity ($\vec{B}$) in (mT) around the MMA, with the highest intensities concentrated across the structure of the MMA. The direction of the magnetic field is represented by the arrow vectors, which indicate its spatial variation. (ii) The horizontal displacement ($D_{m)}$ of the MMA along the x-axis was analyzed numerically and experimentally under external magnetic field strengths (ΔB) ranging from 0 to 5 mT. In the absence of an external magnetic field (ΔB=0 mT), the MMA remained stationary within the microchannel. Upon applying an external magnetic field (ΔB=5 mT), the induced force resulted in a pronounced horizontal displacement of the MMA. The numerical data and experimental measurements are plotted to highlight the correlation between the external magnetic field strength and the resultant displacement. Insets depict the microchannel setup, demonstrating the MMA's static position in the absence of an external field (coil off) and its dynamic response under the influence of a 5 mT field (coil on). The results verified the strong agreement between the numerical predictions and experimental data, confirming the system's reliability in achieving translational motion of the MMA under controlled magnetic field variations.

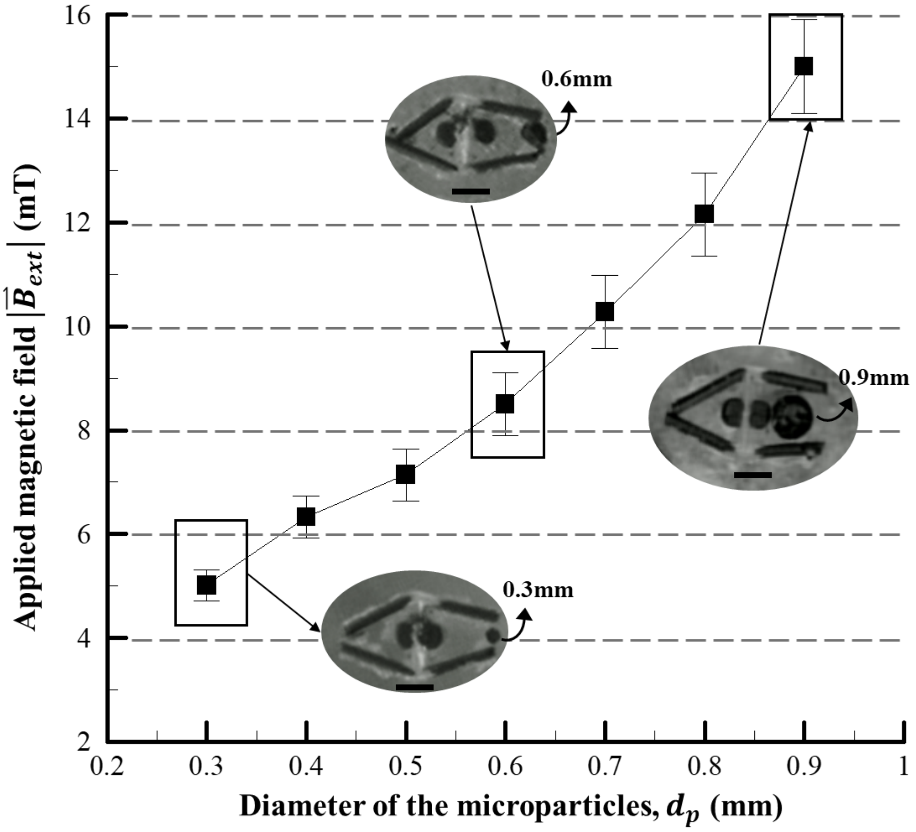


**Figure S4. The versatility of the presented MMA in grasping different sizes of microparticles.** The arms of the presented MMA were controlled to open across distances ranging from 0.3 mm to 1.4 mm, allowing the grasping of particles sized between 0.3 mm and 0.9 mm. A particle measuring 0.3 mm was grasped under an applied magnetic field of 5 mT. To grasp a particle of 0.9 mm, a magnetic field of 15 mT was required. However, at this field strength, significant arm bending can be observed, which could potentially damage both the MMA and the grasped particle by introducing excessive stress. Therefore, it is suggested that the presented MMA is suitable for grasping particles within the range of 0.3 mm to 0.8 mm without causing damage. The scale bar represents 1 mm. Error bars indicate the standard deviation based on three repeated experiments.

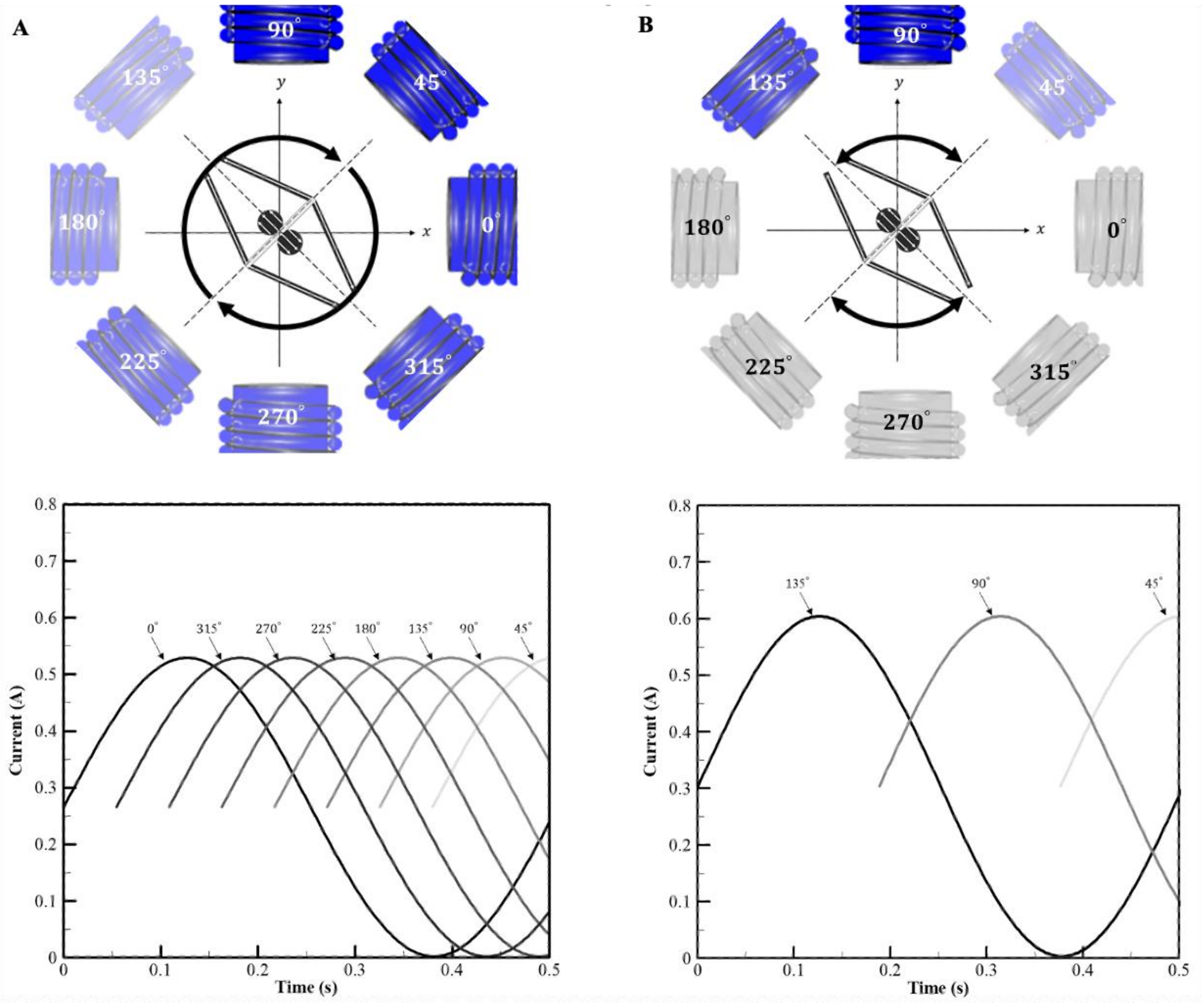


**Figure S5. Phase adjustment in the input current supplied to the EMC system for inducing MMA's rotational and swinging motions.** To enable the rotational motion of the MMA, the phase differences were applied to the input currents supplied to the electromagnetic coil (EMC) system. The phase differences were configured using the Laboratory Virtual Instrument Engineering Workbench (LabVIEW, National Instruments, Austin, TX), allowing real-time modulation of the current supplied to each EMC. The eight EMCs, positioned at angles of 0°, 45°, 90°, 135°, 180°, 225°, 270°, and 315° relative to the MMA, were programmed with specific phase offsets. Sequential activation of the EMCs, based on these phase differences, generated controlled clockwise or counterclockwise rotational motion of the MMA, as depicted in panel (A). In this panel, the EMCs actively contributing to the motion are highlighted in blue, while inactive coils are shown in gray. The corresponding current profile for the full rotational motion, represented as a sine waveform, is displayed below the panel. The applied current for this operation was set to 0.52 A. To achieve the swinging motion of the MMA, only the EMCs positioned at 135°, 90°, and 45° were sequentially activated by employing the same phase control method. This is illustrated in panel (B), where active coils are highlighted in blue and inactive coils remain gray. The sine

waveform representing the current supplied for swinging motion is shown below the panel, with the current magnitude manually set to 0.58 A. The use of distinct colors in the figure visually differentiates the active and inactive EMCs for each motion, providing clarity regarding the magnetic activation sequence.

## 2. Supplementary Text

### 1. The expressions to calculate the MMA arm's magnetic force, torque, stiffness, displacement, and grasping force

This section focuses on the analysis of force, torque, and displacement associated with the MMA's magnetic arms to elucidate the fundamental interaction between magnetic fields and the mechanical response of the MMA arms. In the proposed MMA design (**Figure S6**), each magnetic arm was conceptualized as a cantilever beam, with one end anchored to the actuator's structural framework composed of PDMS, while the opposite end remained free. The arms were fabricated using a composite material consisting of PDMS and NdFeB particles. Upon being subjected to an external magnetic field, the magnetic force was uniformly distributed along the beam due to the homogeneous dispersion of magnetic particles within the composite. This magnetic force was determined using Equation (1)[1].

$$F_m = \nabla(m \cdot B) \qquad (1)$$

And, for a simple case with a uniform field gradient

$$F_m = \mu_0 MV \frac{dB}{dx} \qquad (2)$$

where

$F_m$ is the magnetic force (N, Newtons)

$m$ is the magnetic moment ($A \cdot m^2$, Ampere meter squared)

$B$ is the magnetic field (T, Tesla)

$\mu_0$ is the permeability of free space ($4\pi \times 10^{-7}\ H/m$)

$M$ is the magnetization ($A/m$)

$V$ is the volume of the magnetic material ($m^3$)

$\frac{dB}{dx}$ is the magnetic field gradient ($T/m$)

Simultaneously, magnetic torque was induced in the arms as a result of the interaction between the applied magnetic field and the intrinsic magnetization direction of the arms. The magnetic torque was calculated using Equation (3)[1].

$$T_m = MVBsin(\theta) \qquad (3)$$

The units of $T_m$ is $(N \cdot m)$). The magnitude of the magnetic torque reaches the maximum when the external magnetic field and the internal magnetization vectors get aligned in opposite directions. Conversely, the torque value diminishes to zero when these vectors are oriented in the same direction.

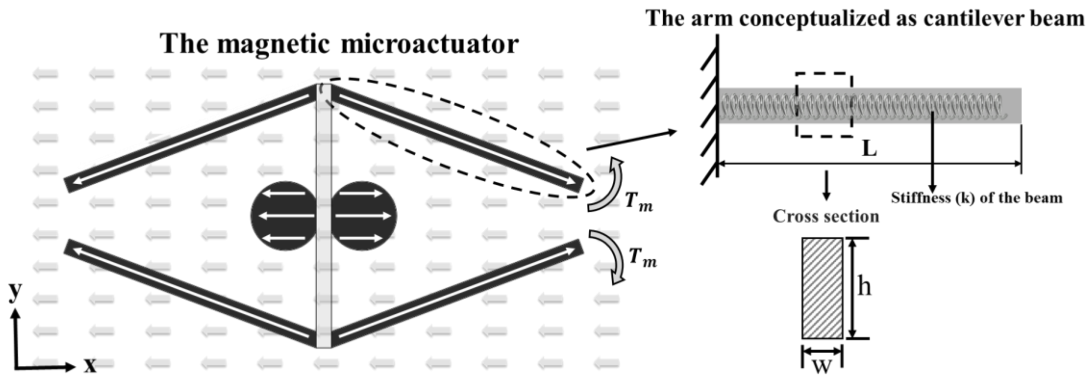


**Figure S6. The arms of MMA conceptualized as the cantilever beam for investigating its structural and mechanical responses.** Upon being subjected to the external magnetic field, the magnetic torque gets induced on the arms, which eventually displaces them to open up for grasping the microparticles or objects. To analyze the mechanical response, the arms were modeled as cantilever beams, with one end fixed to the MMA structure made of PDMS and the other end free. The structural and mechanical responses were then analyzed using analytical expressions derived from beam theory.

Apart from the induced magnetic force and magnetic torque, one of the critical material properties of the arm, such as the stiffness plays an important role in determining the displacement of the arm (the arm opening distance). The stiffness of the magnetic arm (conceptualized as the cantilever beam) can be determined using Equation (4)[2].

$$k = \frac{3EI}{L^3} \qquad (4)$$

The unit of $k$ is $(N/m)$

$k$ is the stiffness $(N/m)$

$E$ is the Young's modulus $(Pa, N/m^2)$

$I$ is the moment of inertia $(m^4)$

$L$ is the beam length $(m)$

The stiffness coefficient approaches zero for extremely soft arms (low E), resulting in infinite flexibility, which may not be suitable for particle grasping. Conversely, for highly rigid materials, such as metals with a high Young's modulus, the stiffness coefficient reaches its maximum, making arm deflection challenging. Therefore, the material properties, including

Young's modulus, along with structural parameters such as the moment of inertia and arm length, should be carefully selected to ensure effective grasping functionality for the intended applications.

By employing a beam theory, the displacement ($\delta$) equation for the magnetic arm can be written as

$$\delta = \frac{T_m L}{EI} \qquad (5)$$

The unit of displacement ($\delta$) is ($m$). The value of $\delta$ reaches the maximum when the applied magnetic torque and the length of the arm are significantly higher with low E. For the same value of magnetic torque, if the arm becomes hard (higher E), then the value of $\delta$ decreases drastically even with a high value of arm length. Therefore, the arm's flexibility and material stiffness must be carefully balanced to achieve the desired displacement, ensuring effective grasping without compromising structural stability.

The gripping force ($F_g$) is a critical parameter in analyzing the performance of MMA that determines its ability to securely hold, manipulate, and release particles of varying sizes. The gripping force of the magnetic arm in MMA can be expressed as the total force exerted at the contact point (between the arm and the particle, **Figure S7**), ensuring a stable grasp under external disturbances. In this analysis, the MMA was assumed to be kept in a static equilibrium state while grasping the particles. Additionally, the dynamic effects, including the inertia of both the actuator and the particles, were neglected, which would otherwise introduce complex transient forces. The gripping force primarily arises from two key components such as elastic restoring force and magnetic force, as shown in **Figure S7**, Equation (6)[1].

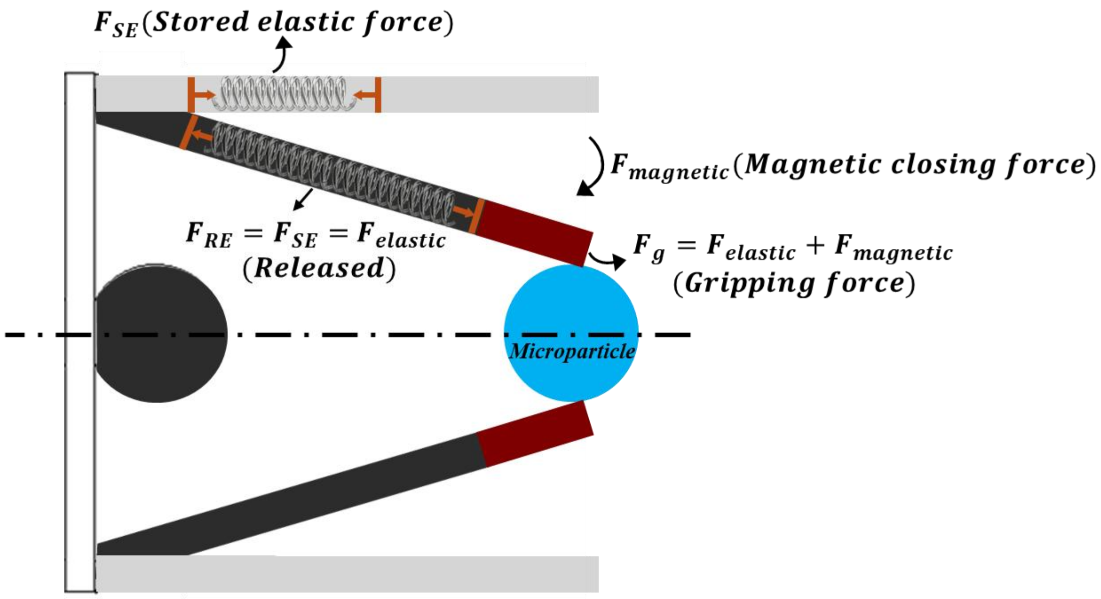

**Figure S7. The schematic illustration of the gripping force induced by the arm during particle grasping.** The total gripping force of the magnetic arm was calculated by considering two critical forces such as the magnetic closing force and the elastic restoring force. The inertial forces of the actuator and particles were neglected to simplify the calculations and avoid unnecessary complexity.

$$F_g = F_{elastic} + F_{magnetic} \quad (6)$$

$$F_{elastic} = k\delta \quad (7)$$

Substituting the expression of k from (4) and $\delta$ form (5) in (7),

$$F_{elastic} = \frac{3EI}{L^3} \times \frac{T_m L}{EI} = \frac{3T_m}{L^2} \quad (8)$$

Now, the expression for $F_{magnetic}$ is known from (2)

$$F_{magnetic} = \mu_0 MV \frac{dB}{dx} \quad (9)$$

Substituting the expressions of $F_{elastic}$ (8) and $F_{magnetic}$ (9) back in (6),

$$F_g = \frac{3T_m}{L^2} + \mu_0 V \frac{dB}{dx} \quad (10)$$

Substituting the expression of $T_m$ from (3) in (10),

$$F_g = \frac{MV}{L^2}\left(3Bsin(\theta) + L^2 \mu_0 \frac{dB}{dx}\right) \quad (11)$$

Thus, the total gripping force can be seen to be a function of the MMA arm's material property (the magnetization), structural dimensions (the volume), and external field parameter (magnetic field), making it a design-critical factor in several applications. Furthermore, the gripping force exerted during particle manipulation was analytically determined using Equation (11), revealing an increase in force with particle size. For instance, a gripping force of 14.3 μN was calculated for grasping a 0.3 mm particle (the required value of the magnetic field is 5 mT), while the force of 42 μN was calculated for grasping the particle of diameter 0.9 mm (the required value of the magnetic field is 5 mT), accompanied by significant arm bending. These findings suggest that the MMA operates most effectively when grasping particles of sizes less than 0.9 mm, balancing sufficient force generation without compromising the structural integrity of the arms or causing damage to the manipulated particles.

## 2. Magnetic field-induced force, torque, and displacement analysis of the magnetic arm and the associated grasping force on the grasped particles

The arm opening distance of MMA plays a crucial role in determining its grasping capability, directly influencing its ability to securely capture and manipulate particles of varying sizes.

Further, understanding this relationship is essential for ensuring effective particle handling while maintaining the structural stability of the MMA during operation. A numerical simulation was performed to estimate the arm opening distance across a range of applied magnetic field strengths, as shown in **Figure S8**. **Figures S8A and S8B** depict the MMA's arm configuration in both closed and open states under the influence of an external magnetic field. Further, it should be mentioned that the pair of arms on each side maintained a predefined separation distance of 0.3 mm. This inherent spacing established the lower threshold for the MMA's grasping capability, limiting the handling of particles or objects to a minimum size of 0.3 mm. Further, the numerical results indicated that the arm opening distance increased with increasing magnetic field intensity, reaching 1.1 mm at 10 mT and expanding further to 1.4 mm at 15 mT, as shown in **Figures S8C and S8D**. This trend was experimentally validated, demonstrating a strong correlation with a margin of error below 5%. Despite the MMA's ability to achieve varying arm opening distances, noticeable arm bending was observed beyond a magnetic field strength of 12 mT, as illustrated in Figure S4. Consequently, the effective range for arm opening was identified as 0.3 mm to 1.2 mm, enabling the grasping of particles with diameters between 0.3 mm and 0.8 mm.

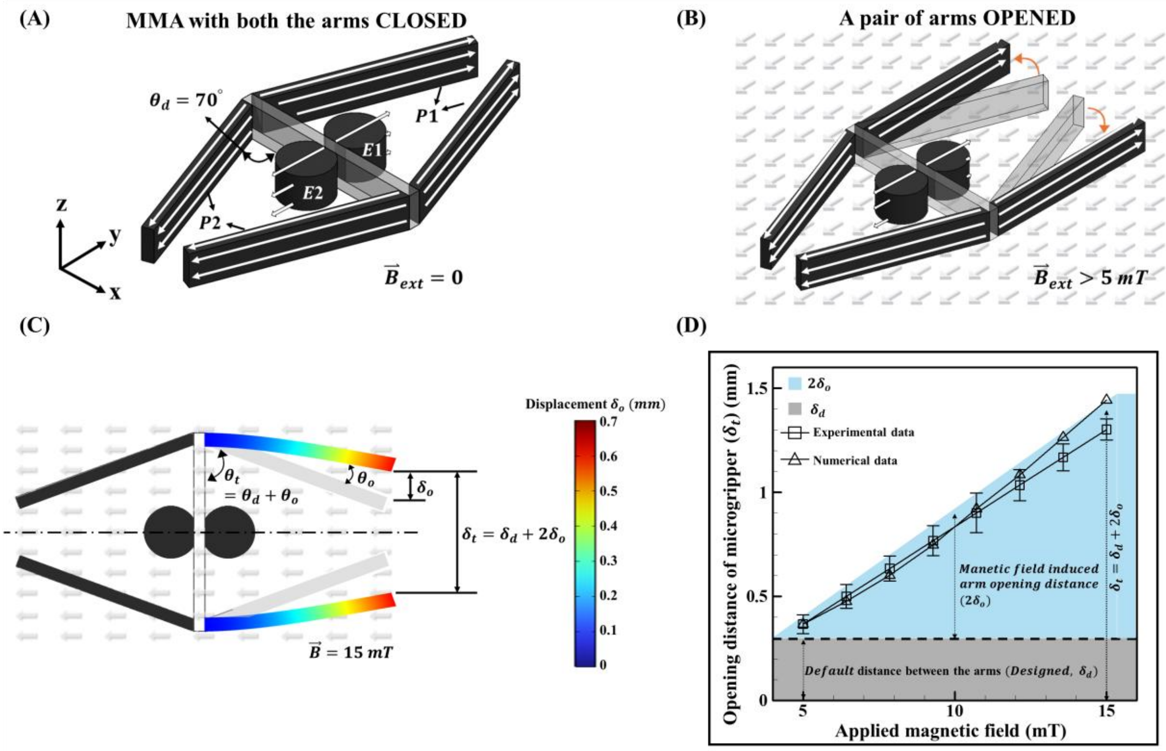


**Figure S8. Analysis of the MMA arm displacement under varying external magnetic fields. (A) and (B)** depict the closed (default) and opened arm configurations of the presented MMA, respectively. For the given MMA design, the arms were predicted to open at a magnetic field value of 5 mT, which was closely aligned with the experimental finding shown

in Figure 2C (ii) of the manuscript. **(C)** The simulated arm opening distance under an external magnetic field of 15 mT is shown. The total arm opening distance is denoted as $\delta_t = \delta_d + 2\delta_o$, where $\delta_t$, $\delta_d$, and $\delta_o$ represent the total, default, and magnetic field-induced arm opening distances, respectively. At 15 mT, the magnetic field-induced arm opening distance $\delta_o$ was predicted to be 0.55 mm, and for a pair of arms, $2\delta_o$ was 1.1 mm. To obtain the total arm opening distance, the default arm opening distance of 0.3 mm was added to $2\delta_o$, resulting in a total of 1.4 mm (1.1+0.3). **(D)** For different values of the magnetic field, the numerical and experimental values of the total arm opening distance are plotted, as shown in Figure S8D. As the magnetic field strength increased, the arm opening distance was observed to increase. For example, at 10 mT, $\delta_t$ was 1.1 mm, and it was increased to 1.4 mm at 15 mT. However, at this maximum value of $\delta_t$, significant arm bending can be observed, as shown in Figure S4. Therefore, such extreme values of arm opening distance should be avoided to prevent potential damage to the MMA arm and to minimize unwanted forces or stress on the grasped particles. Error bars indicate the standard deviation based on three repeated experiments.

**3. Investigation of the MMA's arm performance based on material selection**

The displacement $\delta$ of the MMA arm (the expression is given in Equation (5)) was identified as a critical factor in ensuring effective grasping and controlled actuation. The MMA's performance was primarily governed by its stiffness ($k$), as expressed in Equation (4), which determines the extent of deformation under the influence of magnetic torque. The arm's stiffness can be seen to be dependent on both material properties, particularly Young's modulus ($E$), and structural properties, such as beam length ($L$). While structural adjustments, including variations in beam length, could theoretically alter stiffness, the microscale nature of the proposed system imposed dimensional limitations, rendering substantial changes in beam length impractical. Consequently, the most viable approach to modulate stiffness involved adjusting the material properties, specifically Young's modulus. To comprehensively examine these relationships, an analytical investigation was conducted (using Equations from (3 to 5), exploring different values of Young's modulus, the corresponding stiffness, and the resulting arm displacement.

A range of Young's modulus values was examined to assess the influence of stiffness on the displacement of the MMA arm. For very low values of Young's modulus, such as 0.1 MPa and 1 MPa, the stiffness was calculated to be $1.5 \times 10^{-3}$ N/m and $1.5 \times 10^{-2}$ N/m, respectively,

resulting in displacements of 206.4 mm and 20.64 mm. These excessive displacements exceeded practical limits for controlled microparticle gripping, and the arms' extreme flexibility compromised the restoring force necessary for reliable actuation. Conversely, for high Young's modulus values, such as 1000 MPa, the stiffness reached 15 N/m, causing the displacement to reduce drastically to 0.02 mm. Here, the increased rigidity resulted in a limited arm deformation, preventing effective particle grasping and reducing sensitivity to magnetic actuation. Alternatively, the moderate values of Young's modulus, ranging from 10 MPa to 100 MPa, were calculated to yield a more balanced response. Specifically, at 10 MPa and 100 MPa, the calculated stiffness values were $1.5 \times 10^{-1}$ N/m and 1.5 N/m, with corresponding displacements of 2 mm and 0.2 mm. This range allowed sufficient deformation for microparticle grasping while maintaining enough stiffness to ensure stable actuation and a reliable restoring force.

Based on these findings, a mixing ratio of 4:1 for PDMS and magnetic powder was adopted for the MMA arms, resulting in Young's modulus of 48 MPa. This composition was found to facilitate a displacement suitable for manipulating microparticles and objects. For this material property, the stiffness and displacement were calculated to be 0.6 N/m and a displacement of 0.55 mm, respectively. To further support this selection, the numerically simulated displacement values, presented in Figure S8, were found to closely align with these analytical results. This combination of material properties, together with predefined structural parameters, was shown to facilitate an effective magnetic actuation and controlled arm displacement to ensure consistent grasping performance.

**4. Dye-conveyance efficiency (DCE)**

The flow conveyance functionality of the proposed MMA was demonstrated by introducing 15 µL of methylene blue dye into a microchannel, which had been partially filled with distilled water at a predetermined starting location, labeled as 'I,' as shown in Figure 6C, in the main text. The MMA was initially positioned in close proximity to this location. Subsequently, both rotational and translational motions of the MMA were activated, allowing for the transport of the dye to a target position, denoted as 'O.' The dye transport was facilitated by the drag force generated through the no-slip condition between the dye and the MMA. The entire process was conducted over a duration of 30 seconds, during which the MMA successfully conveyed the dye from position 'I' to position 'O.' To further assess the flow conveyance performance, the MMA's capabilities were evaluated over varying

navigational distances within the microchannel (5, 10, 15, and 20 mm). The DCE was calculated by determining the gray-level deviation from the recorded images using Equations 12 and 13. Specifically, the pixel intensity distribution within the selected region was computed and expressed as the standard deviation relative to the mean intensity value within the region of interest. The calculated value was then subtracted from 1, yielding a final result ranging between 0 and 1. A value of 0 indicated no conveyance, while a value of 1 represented complete dye conveyance.

$$Standard\ Deviation\ (s.d.) = \sqrt{\frac{\sum_{i-1}^{n}(x_i-\bar{x})^2}{n}} \qquad (12)$$

$$DCE = 1 - \frac{s.d.}{x} = 1 - \frac{1}{x}\sqrt{\frac{\sum_{i-1}^{n}(x_i-\bar{x})^2}{n}} \qquad (13)$$

$\bar{x}$ is the average mass fraction across the section
$x_i$ is the mass fraction in the $i_{th}$ cell
$n$ is the number of cells.

**3. Legend for Supplementary Videos S1 to S3**

**Video S1. Sequential grasping of eight microparticles by MMA**
In the video, the function of MMA as the microgripper is demonstrated by sequentially grasping a total of eight particles from their respective positions. The corresponding locomotion strategies of the MMA, along with the coil activation approach, are shown. Initially, the MMA was positioned at the center of the microchannel and was navigated toward various particle locations through the activation of specific coils. For instance, to grasp the microparticle located near 0 degrees, the coil positioned at that angle was supplied with a 0.5 A current, generating a magnetic field gradient of up to 5 mT. This magnetic gradient propelled the MMA toward the target, as shown in the video. To facilitate the grasping function, the supplied current was subsequently increased to 1 A, enhancing the field to 15 mT, which allowed the MMA arms to open. This process was repeated for each particle, enabling all eight microparticles (four on each side of the arms) to be loaded, as demonstrated in the video.

**Video S2. The microassembly task executed by MMA.**
In this video, the sequential loading, transportation, and assembly of two microobjects (M1 and M2) executed by MMA is demonstrated. Initially, the MMA was navigated to the position of the M1 by supplying a current of 0.5 A to the EMC coil positioned at 0 degrees.

Upon reaching the vicinity of M1, the arms were opened by increasing the current to 1.5 A, generating a magnetic flux density of 15 mT. After successfully loading M1, the MMA was navigated back to its initial position and reoriented to approach M2. The arms were opened again to load M2, and the MMA was returned with both objects to their initial position in preparation for assembly. The assembly process began with the MMA navigating to the designated slot for M1, where the arms were opened to release the micro-object. The MMA was then manipulated to align with and navigate to the slot for M2. The M2 was then released by opening the arms and eventually completed the assembly process. The video showcased the precise coordination of EMC activation, MMA navigation, and arm motion for efficient micro-object manipulation.

**Video S3. The flow conveyance and micromixing tasks executed by MMA**

The sequential conveyance and mixing processes are showcased in this video, illustrating the MMA's ability to manipulate fluid flow. In this demonstration, the MMA was actuated to transport three distinct colored dyes, including yellow, blue, and red, within a microchannel, followed by a rotational motion to achieve mixing. Initially, the dyes were conveyed from designated zones A, B, and D to a common target zone C. Once positioned at zone C, the MMA was actuated with a rotational motion to blend the dyes, resulting in the emergence of a black color, demonstrating effective mixing. By the end of the 140-second operation, encompassing both transport and mixing steps, the black color was observed, confirming the MMA's capability for robust dye conveyance and efficient mixing within the microchannel.

***References:***

[1] M. Riad, I. M. Salama, in *Electromagnetic Fields and Waves: Fundamentals of Engineering*, McGraw-Hill Education, **2020**.

[2] B. J. Goodno, J. M. Gere, *Mechanics of materials*, Cengage learning, **2020**.
